# Oldest ctenodactyloid tarsals from the Eocene of China and evolution of locomotor adaptations in early rodents

**DOI:** 10.1186/s12862-018-1259-1

**Published:** 2018-10-04

**Authors:** Łucja Fostowicz-Frelik, Qian Li, Xijun Ni

**Affiliations:** 1 Key Laboratory of Vertebrate Evolution and Human Origins, Institute of Vertebrate Paleontology and Paleoanthropology, Chinese Academy of Sciences, 100044 Beijing, People’s Republic of China; 2 Institute of Paleobiology, Polish Academy of Sciences, 00-818 Warszawa, PL Poland

**Keywords:** Glires, Rodentia, Paleogene, Asia, Foot structure, Locomotion, Morphological evolution

## Abstract

**Background:**

*Tamquammys* has been considered one of the basal ctenodactyloid rodents, which has been documented in the earliest to middle Eocene (~ 56.0–48.5 Ma) in China. It was the most abundant and widespread rodent genus in the Erlian Basin (Nei Mongol, China) and dominated Arshantan small-mammal faunas of that region. Here for the first time we describe the morphology of the astragalocalcaneal complex in *Tamquammys robustus* (larger) and *T. wilsoni*, and interpret it against the background of locomotor adaptations of basal Euarchontoglires (rodents, lagomorphs, tree shrews, and primates).

**Results:**

The comparative morphology of the tarsal elements in *Tamquammys robustus* and *T. wilsoni* shows overall slenderness of the bones and their similarity to the tarsal elements of *Rattus*, a generalist species, and those of small rock squirrels (e.g. *Sciurotamias*). The two species differ slightly in their cursorial ability; smaller *T. wilsoni* shows some adaptations to climbing. The results of principal component analysis of the calcaneus and astragalus support this observation and place *T. robustus* in-between *Rattus* and ground/rock squirrel morphospace, and *T. wilsoni* closer to euarchontans, *Tupaia* and *Purgatorius*.

**Conclusions:**

The morphology of the tarsal elements in *Tamquammys* indicates a generalist rodent morphotype with no particular adaptations to arboreality. We suggest that *Tamquammys* as a basal ctenodactyloid is closer to the ancestral astragalocalcaneal morphology of rodents than that of more derived North American paramyines of similar age. Overall similarity in *Tamquammys* tarsal elements structure to *Purgatorius*, a basal primate, may point to the antiquity of the tarsal structure in *Tamquammys* and a generally unspecialized foot structure in early Euarchontoglires.

**Electronic supplementary material:**

The online version of this article (10.1186/s12862-018-1259-1) contains supplementary material, which is available to authorized users.

## Background

*Tamquammys* is one of the earliest ctenodactyloid rodents known from the early Eocene of Asia [1]. Its remains have been reported from the Eocene deposits of the Zaysan Basin in Kazakhstan [1], the Erlian Basin in Nei Mongol (NE China) [2–4], and the Hetaoyuan Formation, Henan Province in the central China [5]. The earliest species of *Tamquammys* were coeval with the early Eocene rodents *Advenimus*, *Chenomys*, *Cocomys*, and *Yuanomys* [3, 4]), and are preceded in the fossil record by basal simplicidentates, namely Alagomyidae (including *Tribosphenomys*) and Eurymylidae [6, 7]. *Tamquammys* was a relatively long-lasting genus, which survived into the Middle Eocene, matched in this respect only by *Advenimus* [4].

*Tamquammys* was most abundant and diversified in the Erlian Basin, Nei Mongol, where its earliest, although scarce, remains were found, from the upper part of the Nomogen Formation (NS-3), representing the Bumbanian Asian Land-Mammal Age [3, 8, 9]. During the next ALMA, Arshantan, *Tamquammys* became a dominant and, for most of this interval, the only rodent genus in the early Eocene ecosystems of Nei Mongol [4]. Several thousand of specimens (teeth, skull fragments, and postcranial elements) assigned to this genus have been recovered from early-to-middle Eocene fossil beds by screen-washing and surface collecting [3, 4].

**Fig. 1:**
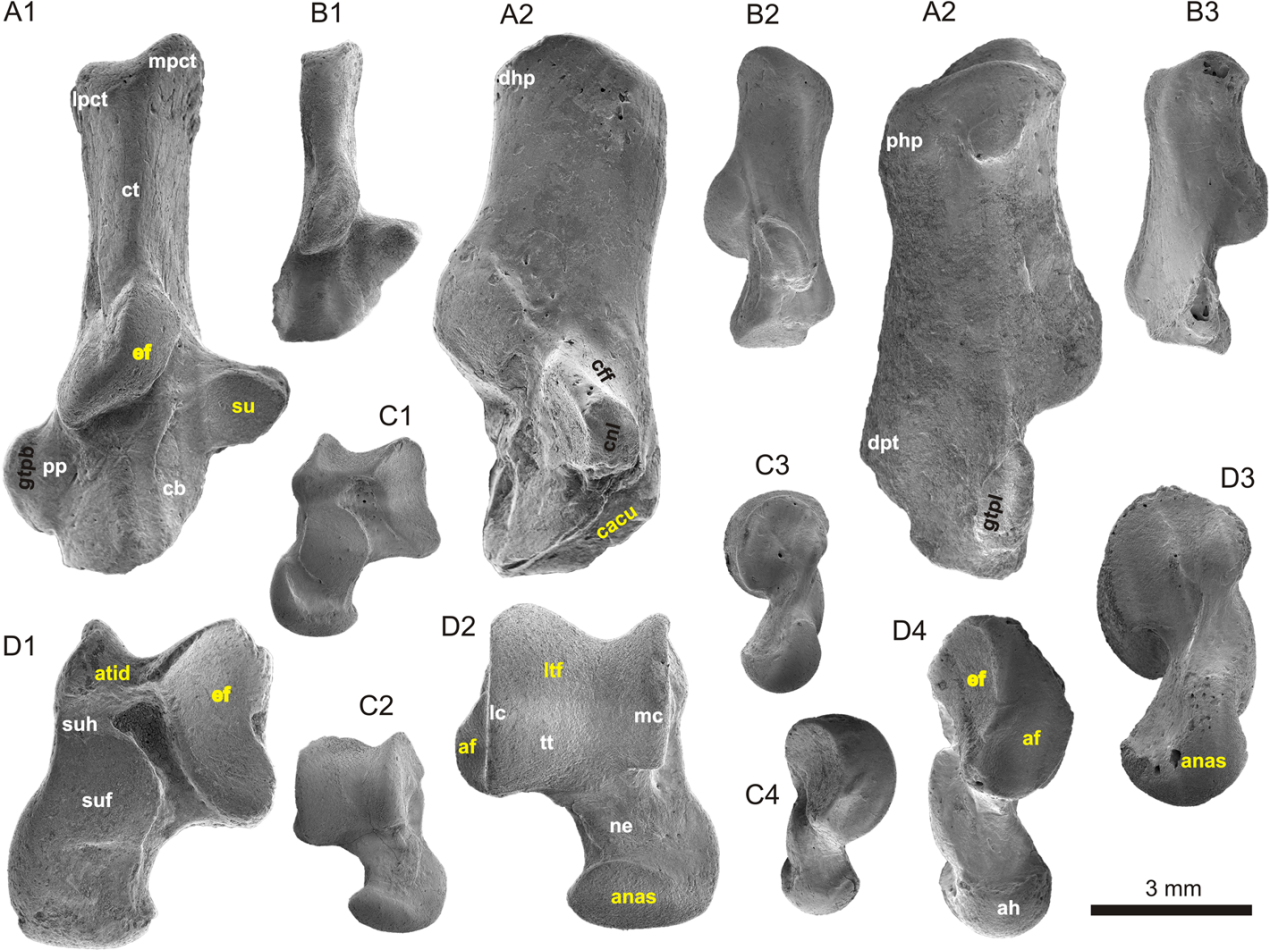
Morphology of calcaneus (**a**, **b**) and astragalus (**c**, **d**) in *Tamquammys robustus* (A, C; IVPP V24136.1 and V24136.4 respectively) and *Tamquammys wilsoni* (B, D, IVPP V V24137.2 and V24137.4 respectively; mirror images) from Nuhetingboerhe, Nei Mongol, China. Bones shown in dorsal (A1, B1, C2, D2), medial (A2, B2, C3, D3), lateral (A3, B3, C4, D4), and plantar (D1, C1) views. Abbreviations (yellow denotes articular surfaces, white other major anatomical structures, and black, grooves for major tendons): af, astragalofibular articulation surface; ah, astragalar head; anas, astragalonavicular articulation surface; atid, distal astragalotibial articulation surface (aff, astragalar groove for tendon of flexor fibularis muscle sensu Chester et al. 2015); cacu, calcaneocuboid articulation facet; cb, calcaneal body; cff, calcaneal groove for tendon of flexor fibularis muscle; cnl, calcaneonavicular ligament; ct, calcaneal tuber; dhp, dorsal heel process; dpt, distal plantar tubercle; ef, ectal facet (on both calcaneus and astragalus); gtpb, grove for tendon of peroneus brevis muscle; gtpl, grove for tendon of peroneus longus muscle; lc, lateral astragalar crest; lpct, lateral process of calcaneal tuber; ltf, lateral tibial facet; mc, medial crest of astragalar trochlea; mpct, medial process of calcaneal tuber; ne, astragalar neck; php, plantar heel process; pp., peroneal process; su, sustentaculum tali with sustentaculum facet; suf, sustentacular facet of astragalus; suh, sustentacular hinge; tt, trochlea tali

Four species of *Tamquammys* have been recognized in the Nei Mongol deposits thus far; *Tamquammys wilsoni* (Fig. 1b, c) was the most abundant and persistent in the fossil record, ranging from the earliest Eocene (Bumbanian) to the early middle Eocene (Irdinmanhan) [3]. *Tamquammys robustus* (Fig. 1a, d), the largest and earliest species of the genus, is known from the early Bumbanian to early Arshantan only. In the earliest Arshantan it coexisted with *T. longus*, which is not known during the Irdinmanhan. In the early middle Eocene (Irdinmanhan) another species, *T*. *fractus*, coexisted with *T. wilsoni*, both being relatively small.

The astragalus and calcaneus are the largest of the ankle bones (tarsals) and form the lower ankle joint, while contributing to the upper ankle joint (the astragalus) and transverse tarsal joint (both bones). The significance of these bones for paleobiology stems from the fact that they are very commonly preserved in the fossil record, second in this respect only to the teeth. The tarsal bones, as a functional unit, are in direct contact with the substrate and thus are immediately related to an animal’s lifestyle [10–12]. Their morphology provides information on the locomotor adaptations of fossil representatives which are otherwise known very incompletely [13, 14]. However, the tarsals of the Eocene Glires have been subject of detailed studies very rarely [15–17].

**Fig. 2:**
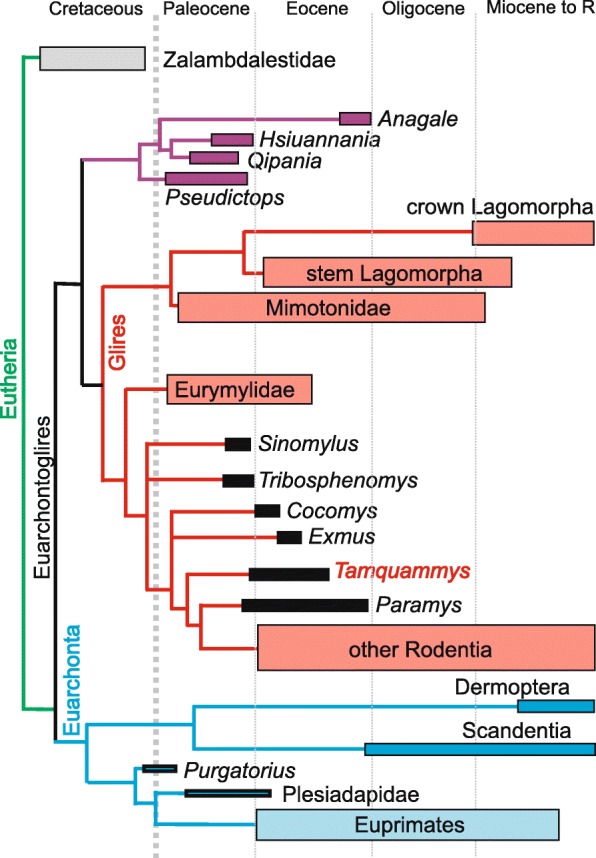
Schematic phylogeny showing relationships between major clades of Euarchontoglires. Note *Tamquammys* position at the base of Rodentia branch. The phylogeny based partly on Li and Meng (2015), and Vianey-Liaud and Marivaux (2016)

This paper focuses on the tarsal bones morphology of the two most abundant species of *Tamquammys* in Nei Mongol: *T. robustus* and *T. wilsoni* (Fig. 1). We compared the bone morphology of *Tamquammys* with that of several genera of Euarchontoglires of known locomotor adaptations in order to establish probable habits of this early rodent. Given that in the recent phylogenies ctenodactyloids are nested below ischyromyid (including paramyine) rodents (Fig. 2), we propose that the former would represent a more basal morphotype than the latter. Our study aims at the reconstruction of supposed locomotor adaptations and lifestyle inference of *Tamquammys* and consequently the probable ancestral morphotype for early rodents. We argue that the putative ‘protorodent’ of Szalay [15] would be rather a generalist than arboreal (scansorial).

## Results

### Comparative morphology of the astragalocalcaneal complex in *Tamquammys*

#### Calcaneus

**Fig. 3:**
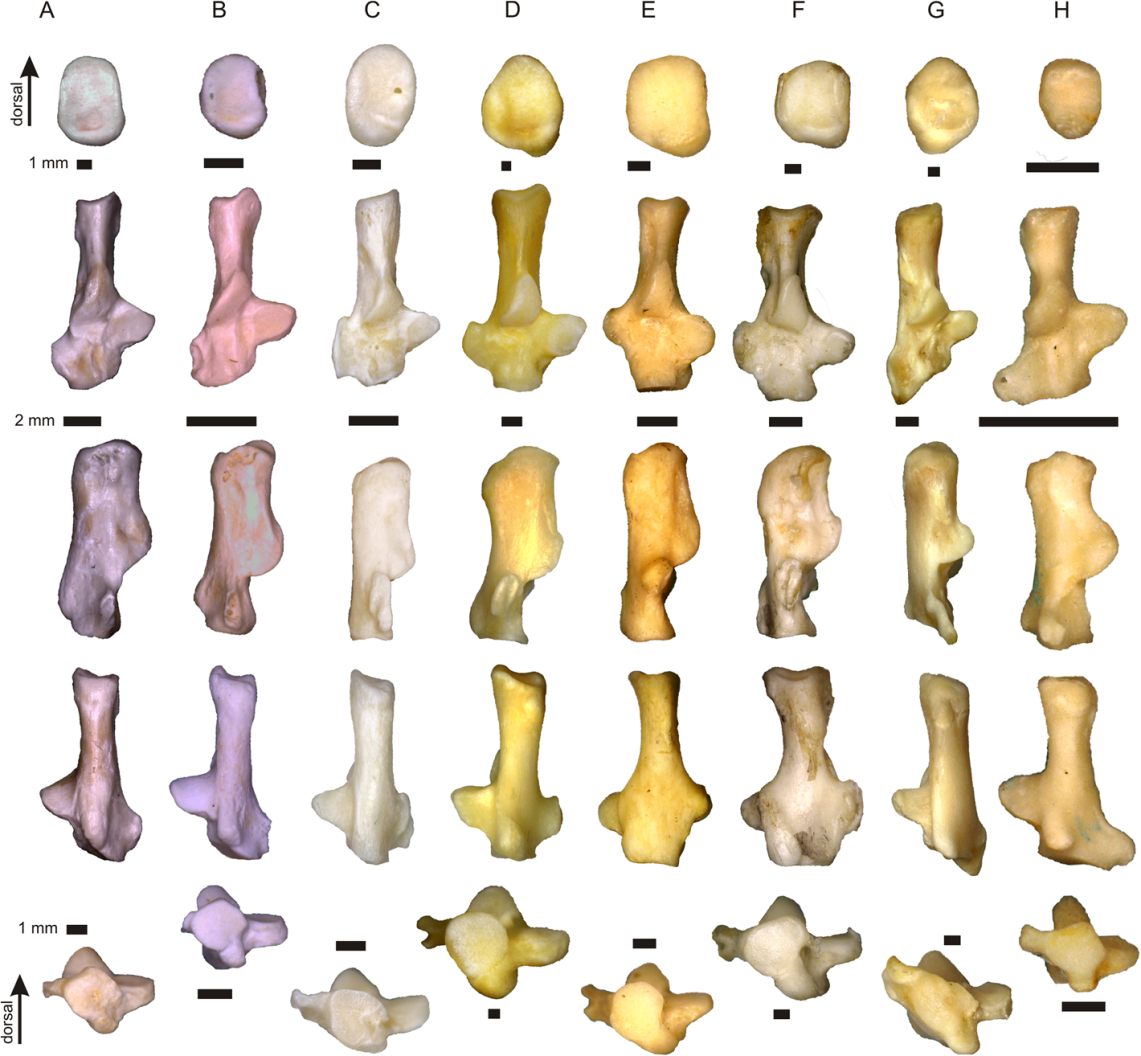
Comparative morphology of the calcaneus (right). *Tamquammys robustus* (**a**) IVPP V24136.3, *T. wilsoni* (**b**) IVPP V24137.1, extant rodents: *Rattus norvegicus* (**c**), *Marmota marmota* (**d**), *Sciurotamias davidianus* (**e**), *Cynomys ludovicianus* (**f**), *Ondatra zibethicus* (**g**), and Paleocene rodentiaform *Tribosphenomys minutus* (**h**, left bone, mirror view). From top to bottom: caudal, dorsal, lateral, plantar, and cranial views

**Fig. 4:**
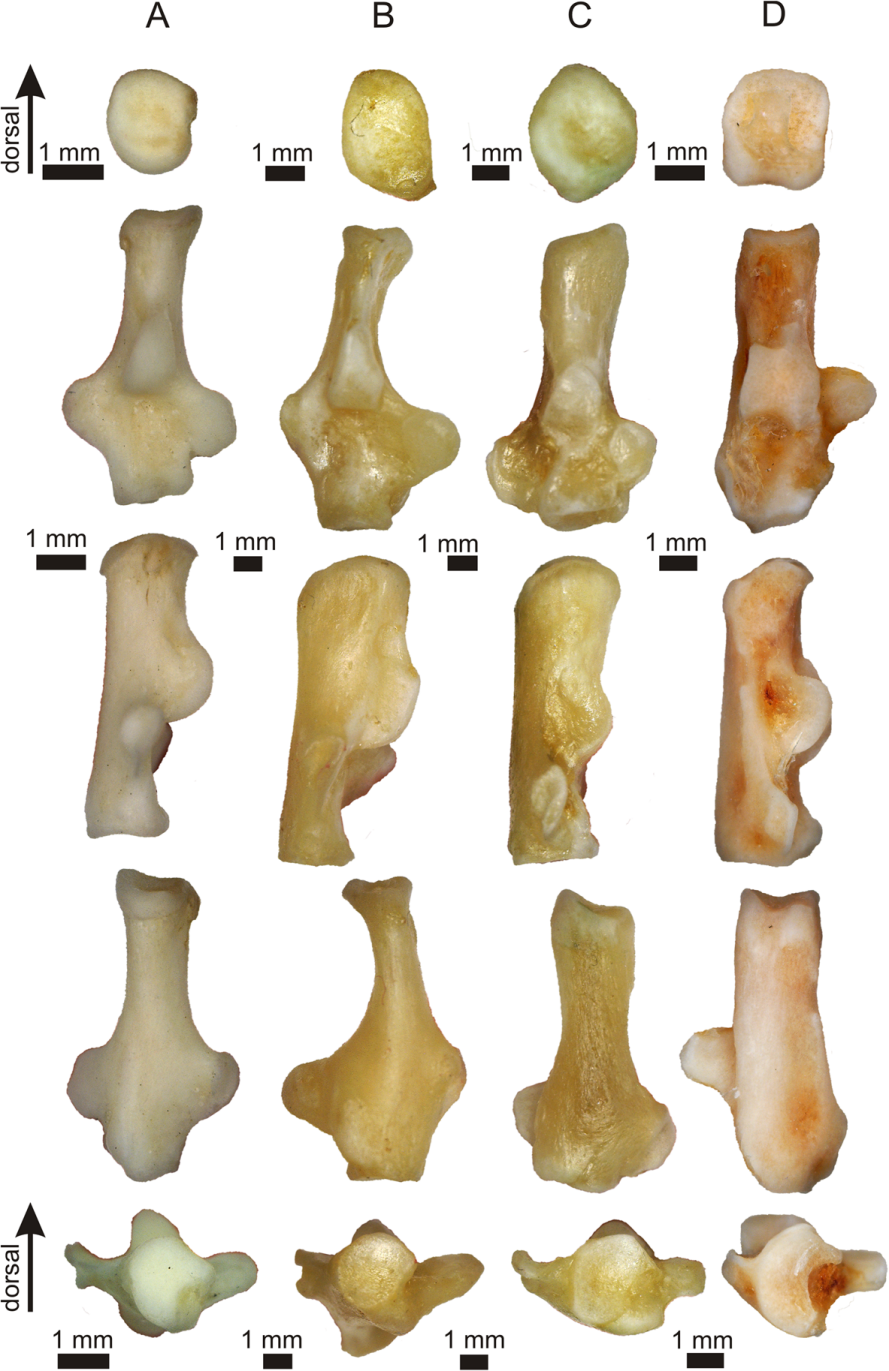
Comparative morphology of the calcaneus (right) continued. Extant rodents: *Glis glis* (**a**), *Sciurus vulgaris* (**b**), *Cricetus cricetus* (**c**), and lagomorph *Ochotona pallasi* (**d**)

The calcaneus of *Tamquammys* is relatively slender (Figs. 1 and 3, Additional file 1: Figure S2A), similarly to that of some ground squirrels (*Marmota* in particular), *Rattus* (Fig. 3), and *Mus*, indicating the overall locomotor dexterity of an animal. The calcaneus of *Tamquammys* is straight, without bending observed in some arboreal rodents (e.g., *Glis*, *Sciurus* see Fig. 4, or *Ratufa*, [12]: fig. 3d; [18]: figs. 12–13), primates (e.g., *Proteopithecus* or *Callicebus*; [19]: figs. 1–3, 12, 13) or to a lesser extent in ground and rock squirrels (e.g., *Cynomys* and *Sciurotamias*, respectively; Fig. 3).

The calcaneal body is moderately elongated (Additional file 1: Figure S2B), which generally reflects aptness to bounding, jumping and swift running [10, 12], and the elongation is greater than that in paramyines (see [17]), *Gomphos*, basal primates (*Purgatorius*), and *Tupaia*, but lesser than in squirrels and the small lagomorph *Ochotona*. The calcaneal load arm is very similar in both species of *Tamquammys* extending between that of *Rattus*, *Cricetus*, and *Marmota*, but closer to the rat (Additional file 1: Figure S2B).

**Fig. 5:**
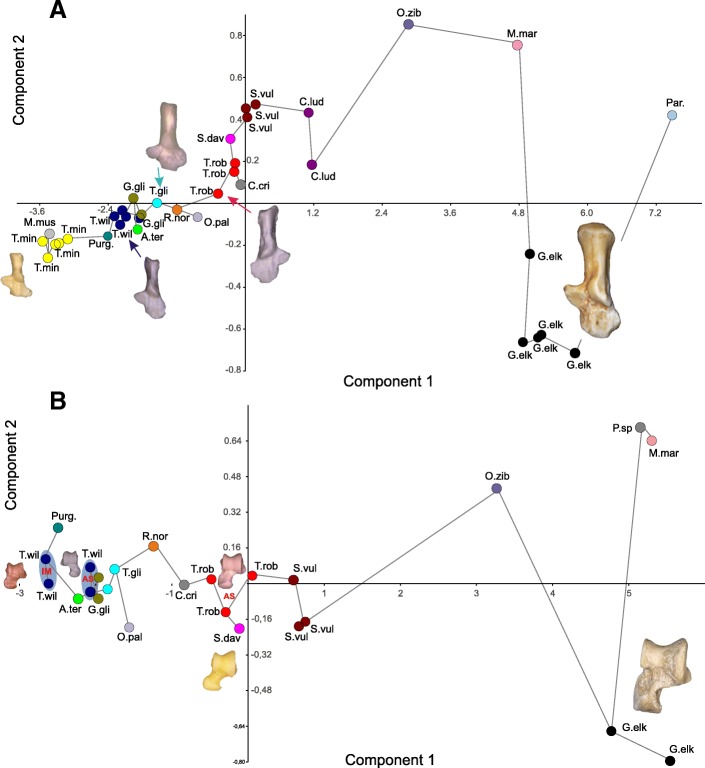
Results of principal component analysis of 15 calcaneal (**a**) and 12 astragalar (**b**) measurements for 19 species. A set of lines connecting all data points represents a minimum spanning tree based on a Euclidean distance matrix. Results support *Tamquammys robustus* as an agile terrestrial ecomorph and *T. wilsoni* as a non-specialized occasionally arboreal species. Species abbreviations: A.te, *Arvicola terrestris*; C.cr, *Cricetus cricetus*; C.lu, *Cynomys ludovicianus*; G.el, *Gomphos elkema*; G.gl, *Glis glis*; M.ma, *Marmota marmota*; M.mu, *Mus musculus*; O.pa, *Ochotona pallasi*; O.zi, *Ondatra zibethicus*; Par., paramyid; P.sp., *Paramys* sp.; Pur., *Purgatorius*; R.no, *Rattus norvegicus*; S.da, *Sciurotamias davidianus*; S.vu, *Sciurus vulgaris*; T.gl, *Tupaia glis*; T.mi, *Tribosphenomys minutus*; T.ro, *Tamquammys robustus*; T.wi, *Tamquammys wilsoni*. AS, Arshantan ALMA; IM, Irdinmanhan ALMA

The sustentaculum tali is relatively large (almost as large as in *Tribosphenomys*, which shows the largest sustentaculum among the studied taxa), roundish in outline and slightly pointed medially, less so than in *Rattus* (Figs. 1 and 3). A large articular surface of the sustentaculum allows for a greater freedom of movement along the astragalocalcaneal juncture in many planes, other than the parasagittal one [12]. The sustentaculum is anteroposteriorly longer than in *Rattus*, similar to that of *Marmota* but wider mediolaterally. The sustentaculum forms an approximately right angle with the calcaneal tuber, and is not inclined anteriorly as in *Cynomys*, *Glis*, *Sciurotamias*, and *Sciurus*. It is placed relatively posteriorly to the calcaneal eminence which bears the ectal facet (Figs. 1 and 3), and its posterior margin is aligned with the mid-length of the ectal facet, similar to *Purgatorius* (see [20]: fig. 1) and *Tribosphenomys*, and only slightly more anterior than in *Gomphos* (and other duplicidentates, showing most posteriorly shifted sustentacula, see e.g., *Ochotona*: Fig. 4d). This is a more posterior position of the sustentacular facet (in relation to the ectal facet) than that in ground and rock squirrels (especially *Cynomys* and *Sciurotamias*), paramyines, and *Tupaia* (see also Fig. 5). The medial edge of the sustentaculum bears a well-shaped groove (Fig. 1) for the calcaneonavicular ligament [21].

In two of the three specimens of *Tamquammys robustus* and in two of five of *T. wilsoni* there is a minute foramen in the calcaneal body at the base of the sustentaculum tali, which may or may not be homologous with the anterior opening of the calcaneal canal in Lagomorpha (sensu Bleefeld and Bock [22]; see also Zhang et al. [23]).

The calcaneal eminence in *Tamquammys* bearing the ectal facet is relatively long (Additional file 1: Figure S2D) and not strongly curved (Fig. 3), again indicating some greater mobility at this surface, especially in the smaller species (*T. wilsoni*). The ectal facet is also somewhat helical (sensu Rose and Chinnery [17]), which suggests improved capability of foot inversion and eversion in *Tamquammys*.

The peroneal process is located in the mid-length of the calcaneal body in *T. robustus* and shifted slightly more posteriorly in *T. wilsoni* (Figs. 1 and 3), and provides a leverage for the tendon of the peroneus longus muscle (running along the lateral surface of the process in a sinusoidal groove, Fig. 1). In *Tamquammys* it is a distinct flattened tubercle, somewhat round at its lateral rim, less extended anteroposteriorly than that in *Rattus*, *Glis*, and smaller than in squirrels such as *Cynomys* or *Sciurus* (Fig. 3), as well as *Purgatorius* ([20]: fig. 1), where it forms a petal-like extension at the calcaneal body. On the other hand, the peroneal tubercle in *Tamquammys* is larger, more flat and shifted more anteriorly than the peroneal tubercle in *Sciurotamias* and other ground squirrels (Fig. 3). The shape of the peroneal tubercle in *Tamquammys* resembles more closely that of paramyines; in *T. wilsoni* it is slightly closer to the calcaneocuboid surface than in *T. robustus*.

The anterior plantar tubercle is distinct in *Tamquammys* (better developed than in *Rattus*), forming a round knob at the plantar surface of the bone, roughly in the sagittal plane of the bone (not shifted medially as in some ground squirrels, Fig. 3e, f). It is located quite far from the edge of the calcaneocuboid surface (but relatively closer than in Duplicidentata; [24, 25]). In both species of *Tamquammys* the plantar surface of the calcaneus between the anterior plantar tubercle and the plantar heel process is gently concave, similar to that of ground squirrels (especially *Marmota*) and *Tribosphenomys* (Fig. 3).

The calcaneal tuber in *Tamquammys* is moderately long and slender, compressed mediolaterally and high dorsoplantarly (Figs. 1 and 3). The caudal surface of the tuber, forming the insertion for the Achilles tendon is regularly oval in *T. wilsoni* and rectangular in *T. robustus* (Fig. 3). The groove marking the tendon attachment is oriented mediolaterally, indicating the pull of the calcaneal tendon exerted strictly in the parasagittal plane. The shape of the caudal surface of the tuber resembles that of *Rattus*, but is more rectangular and generally more compressed mediolaterally than in all ground squirrels, and much more than in *Gomphos*, which shows a very strong compression of the tuber in the opposite, dorsoplantar direction (Fig. 5).

The medial and lateral tubercles at the calcaneal tuber, which form attachments for the superficial digitor flexors, are well developed and extend strictly in the caudal direction, not flaring mediolaterally as much as in ground squirrels. This supports the observation that the action of these tendons, flexion of the toes, is exerted in parasagittal plane, thus the toes are grasping, but without spreading out very much.

The plantar side of the calcaneal tuber bears a moderately shaped plantar heel process for the origin of flexor digitorum brevis muscle (Fig. 1; [12]: fig. 1a), which is weaker and more terminally located than in ground and rock squirrels, but better developed than in *Rattus* and *Ondatra*. The dorsal side of the tuber in *Tamquammys* is not ‘pinched’ as in typical arboreal taxa (e.g., *Sciurus*, see Fig. 4b, also Rose and Chinnery [17]: fig. 13) and the pit for the calcaneofibular ligament is relatively shallow and placed close to the calcaneal eminence, suggesting that a weak ligament binding rendered the joint relatively flexible.

The calcaneocuboid surface is relatively isometric (Fig. 3, Additional file 1: Figure S2E), roundish to semicircular. The intrageneric difference is slight, but indicates a greater freedom of movement along the calcaneocuboid facet (in mediolateral direction) in *T. wilsoni*, which supports somewhat more developed climbing ability in this species in comparison with *T. robustus*. Furthermore, the surface of the calcaneocuboid facet is inclined ca. 30–40° to the longitudinal plane, which is typical of terrestrial taxa [15, 20]. Such an inclination of the calcaneocuboid surface actually prevents extensive inversion and eversion of the foot. The inclination of the calcaneocuboid surface in *Tamquammys* is stronger than in all studied rodents, including paramyines [17]. The inclined astragalocalcaneal surface is present in *Tribosphenomys* (Fig. 3), *Protungulatum*, *Procerberus*, *Plesiadapis* [21], and *Purgatorius* [20], although the inclination is less marked than that in *Tamquammys*. On the other hand, the stronger inclination is found in all duplicidentates (e.g., in *Gomphos*, *Mimolagus*, and lagomorphs (Fig. 4d); [26, 27]), which are cursorial.

#### Astragalus

**Fig. 6:**
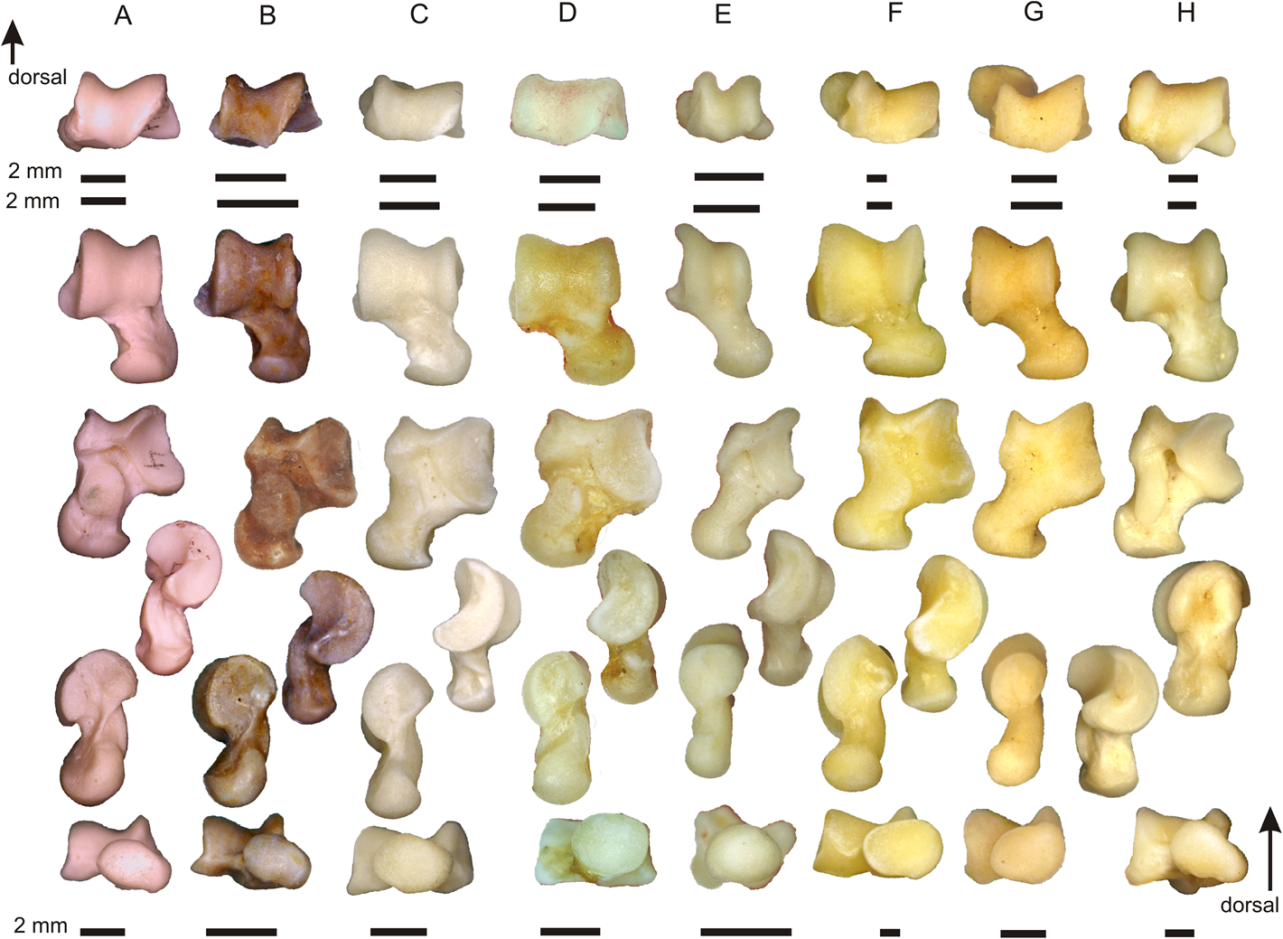
Comparative morphology of the astragalus (right). *Tamquammys robustus* (**a**) IVPP V24136.4; (**b**) *T. wilsoni*, IVPP V24137.5 (mirror image); extant rodents: *Rattus norvegicus* (**c**), *Cricetus cricetus* (**d**), *Glis glis* (**e**), *Marmota marmota* (**f**), *Sciurotamias davidianus* (**g**), *Ondatra zibethicus* (**h**). Note a relatively deep trochlea and long erect neck, both indicators of improved orthal movement

The astragalus of both species of *Tamquammys* is relatively slender with an elongated neck corresponding to a relatively elongated body of the calcaneus (Fig. 6, Additional file 1: Figure S3A and B). The elongation of the neck is lesser than in *Sciurotamias*, similar to that of *Paramys* sp. [17] and *Tupaia*, but larger than in *Rattus*, *Marmota*, and *Purgatorius* (Fig. 3, Additional file 1: Figure S3B).

The trochlea is deep and slightly asymmetric, deeper than in *Rattus*, *Ondatra* (Fig. 6), primates, and *Tupaia*, with the well-defined and high ridges, which are, however, lower than those of *Glis*. The trochlear area of the lateral tibial facet (*ltf* sensu Chester et al. [20]) and especially the distal astragalotibial surface [15] (astragalar groove for the tendon of the flexor fibularis muscle sensu Chester et al. [20]) are extended anteroposteriorly, suggesting a considerable anteroposterior freedom of movement in the crural joint. On the other hand, the lateral tibial facet at the trochlea does not extend at the astragalar neck, unlike in some basal primates where it is related to climbing adaptations [20], but ends at the trochlea-neck contact as in other rodents studied here (Fig. 6). The trochlea does not slope mediolaterally, unlike in *Purgatorius* (and other basal Euarchonta, see Chester et al. [20]: figs. 1 and 3), which supports at least limited cursorial adaptations in *Tamquammys*, and the absence of typical arboreal traits.

The neck of astragalus in *Tamquammys* is relatively erect and long, aligned closely to the sagittal axis of the bone, what can also be interpreted in favor of increased cursorial abilities, as such neck structure is typical of cursorially adapted rodents [12] and duplicidentates [25, 26]).

The astragalar head is strongly compressed dorsoventrally (but less than in paramyines and *Purgatorius*). In distal view the head is oval, slightly pear-shaped (wider laterally), and has a posteriorly extended articular facet for the navicular (Figs. 1 and 5), which encroaches onto the neck (higher than in most of the analyzed rodents except for *Ondatra*, and higher than in *Tupaia*), implying a great range of rotation in this joint, thus increased maneuverability of the foot. The astragalonavicular surface in *Tamquammys* is more convex than in *Rattus*, ground and rock squirrels, and *Tupaia*, although it is not as much bulged medially as in *Purgatorius* and plesiadapids.

The head is slightly rotated at the neck (ca. 20° dorsally); a similar rotation is observed in *Paramys* (see Rose and Chinnery [17]: fig. 11c), *Ondatra*, and *Gomphos*, whereas in ground squirrels, *Rattus*, *Tupaia* (Fig. 5), and primates (e.g., *Purgatorius* and plesiadapids) the long axis of the head is oriented in mediolateral direction.

The astragalar ectal facet is subtriangular in outline (the wider side directed caudally) and concave. It is similar to that of *Rattus* and ground squirrels, but shallower than in *Ondatra*. The astragalar sustentacular facet is regularly oval (elongated craniocaudally). In *Tamquammys robustus* it is not fused or contacting the astragalonavicular facet (Figs. 1 and 6). In *T. wilsoni* the sustentacular facet is less defined posteriorly and somewhat extended towards the sustentacular hinge (Fig. 1 C1), although it does not form a complete smooth extension onto this surface as it does in sciurids, paramyines [17], *Ondatra*, *Tupaia*, and in the plesiadapiform *Dryomomys*. Such extension is, however, absent in *Purgatorius* (see Chester et al. [20]: fig. 3d-e) and in *Gomphos* [24].

The astragalofibular facet at the medial wall of the medial crest of the astragalar trochlea is gently semicircular and concave (Figs. 1 and 6). The surface of the facet is upturned somewhat dorsally and has relatively larger area in both species of *Tamquammys* than that in *Rattus* and ground squirrels, resembling the facet of *Ondatra*, which is wider and more tightly curled (twisted). The facet serves as a strong support for the fibula, especially during the foot eversion, blocking the fibula from ‘slipping’ laterally or plantarly. Such dorsally upturned fibular surface is not present in typically cursorial animals (e.g., in lagomorphs [25]), but can be observed in *Glis,* which is an arboreal animal with good climbing ability. The facet is also not extended dorsally in *Cynocephalus*, *Ptilocercus*, and *Tupaia*, in contrast to that in *Notharctus* (Chester et al. [20]: fig. 3f) and some anthropoids [19]. The astragalofibular facet apparently serves as a temporary support for the fibula coming dynamically into action, especially during the foot eversion. It may allow stabilizing the crural joint during swift movements on uneven branches or terrain, when some additional ankle support is required. An upturned astragalofibular facet is lacking in *Cynocephalus*, because this animal employs a different mode of locomotion (crawling and claw climbing upon vertical substrates, or hanging beneath the branches). Given its big size, the colugo requires a weight bearing mechanism that would stabilize the foot, and the forces acting on the animal’s limb bones are directed quite differently to these in e.g. *Glis*.

### Locomotor adaptations in *Tamquammys*

The morphology of the astragalocalcaneal complex of *Tamquammys* does not indicate any prevailing locomotor adaptations, but rather suggests this animal was an agile terrestrial-scansorial generalist (Fig. 5).

The elongated, helical and not tightly curled ectal facet of the calcaneus, deep, posteriorly extended, tibial facet of the astragalus, relatively strong and large peroneal process, and extended astragalonavicular facet at the astragalus head indicate great mobility of the foot, especially its dorsoplantar flexibility at the crurotarsal joint [20]. Such potential for movement range is also indicated by a well developed and large sutentacular facet. Large (especially wide) sustentacula in rodents allow for greater freedom of movement along the astragalocalcaneal juncture and, according to Ginot et al. [12], are associated with increased climbing or aquatic adaptations, requiring foot to move effectively not simply in the parasagittal plane. The facilitation of sliding along the articular surfaces (e.g., not profoundly convex ectal facet) at the astragalocalcaneal contact points to efficient eversion–inversion of the foot [20]. This is especially true for the smaller species, *Tamquammys wilsoni*, although the extent of such foot rotation must have been much more limited in both species of *Tamquammys* than in typically arboreal taxa.

On the other hand, the structure of the crurotarsal surface reflected by a deep trochlea with straightly arranged ridges stabilizes the tibial insertion into the joint (protects against dislocations) and makes the joint more restricted in terms of the freedom of the mediolateral movements at the articulation surfaces, and can be regarded as cursorial adaptations (perfected e.g., in lagomorphs [25], but observed also in cursorial rodents [12]). This morphological trait in the astragalocalcaneal complex of *Tamquammys* is also supported by the relative slenderness and somewhat elongated calcaneal body and relatively erect astragalar neck. The inclined calcaneonavicular surface of the calcaneus also prevents a large degree of rotation at this surface, thus the foot position is more stable.

The elongation of the calcaneal body in Euarchonta is correlated with an increase of the jumping ability [10], be it terrestrial leaping or arboreal vertical jumping (but see also Moyà-Solà et al. [28] for a different explanation); in the latter case the elongation of the calcaneal body can be brought to extreme as in e.g., *Tarsius* or Omomyidae [19, 20]. The same regularity, although to a much lesser extent was reported also for Glires; e.g., in highly cursorial leporids [25] and some rodents (e.g., Ginot et al. [12]), indicating the proximal calcaneal elongation as one of the hallmarks of cursorial/or jumping Euarchontoglires. The relative elongation of the calcaneal body in *Tamquammys* indicates moderate jumping/bounding potential, probably better developed than in *Rattus* and *Tupaia*, but less in comparison to rock or ground squirrels (especially smaller genera, such as *Sciurotamias* and *Cynomys*), which display increased bounding.

**Fig. 7:**
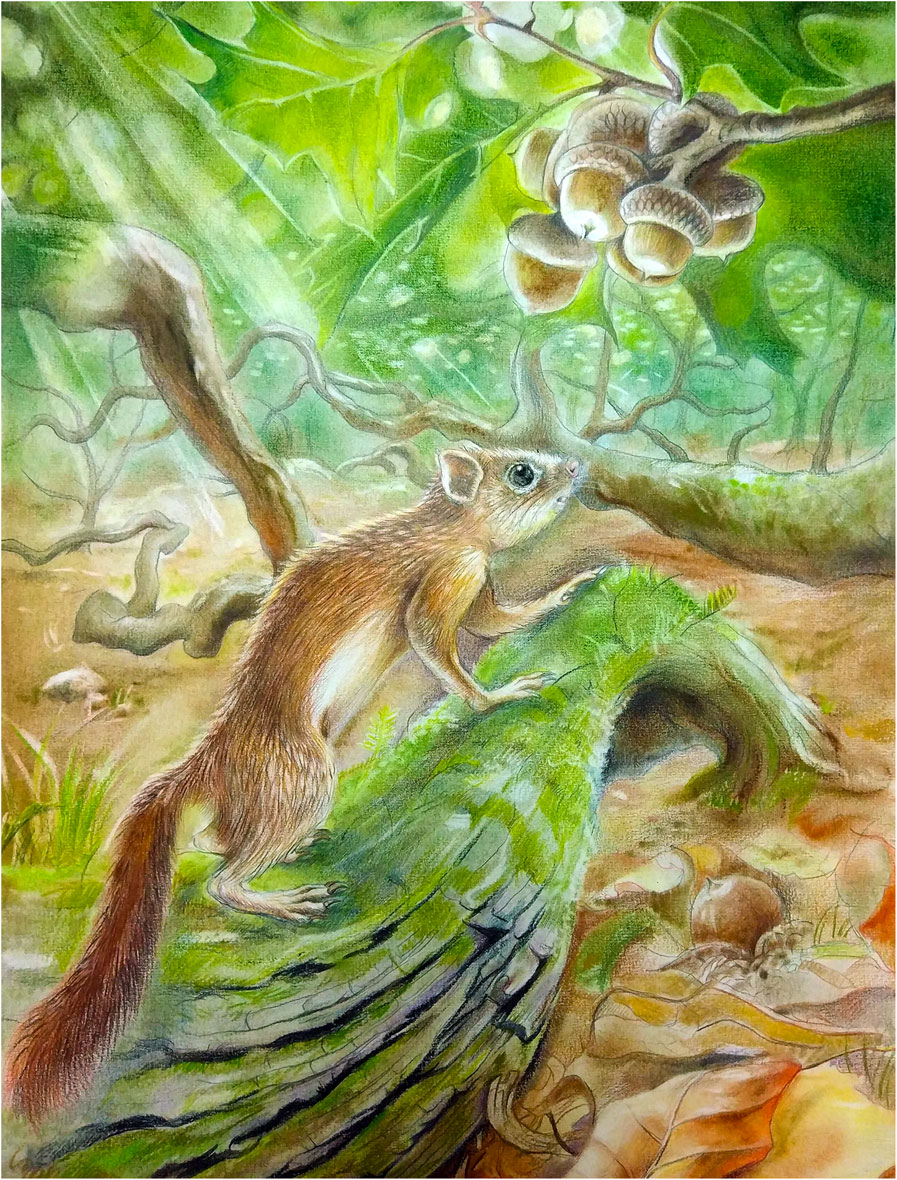
Life reconstruction of *Tamquammys robustus* in a forest environment during the early Eocene (Arshantan) in the Erlian Basin (Nei Mongol, China). Hand drawing by Xiaocong Guo

Judging on the skeletal traits presented here and supported by paleoenvironmental data [7, 9, 29] we propose *Tamquammys* as a forest floor dweller, occasionally climbing at the lower branches (Fig. 7), in search of food or escaping from predators, capable also of a swift run on uneven terrestrial substrate. The overall greater slenderness, longer helical ectal facet, and slightly longer calcaneal body in *T. wilsoni* suggest that it was more likely climbing trees or shrubs than larger *T. robustus*. The results of principal component analysis (Fig. 5; Additional file 1) of the astragalocalcaneal complex (especially of the calcaneus) support such interpretation, with *Tamquammys robustus* positioned in morphospace closer to *Rattus* and ground/rock squirrels (*Cynomys* and *Sciurotamias*), and *Tamquammys wilsoni* clustering with *Purgatorius* and *Tupaia*, showing basic arboreal adaptations (Fig. 5).

### Principal component analysis

#### Calcaneus

Results of principal component analysis include calcanei attributed to *Tamquammys robustus* plotting within generally cursorial to terrestial rock climbing species morphospace, which is distinctly separated from typical ground squirrels and large taxa including semiaquatic *Ondatra* (Fig. 5a). *T. wilsoni* plots closest to *Glis glis* and near unspecialized arboreal species morphospace. Principal component 1 (PC1) represents 96.6% of the variance. All variables show positive loading for PC1 (Additional file 1: Figure S4) and they are weakly positively correlated (0.22–0.32) with this component. Generally, a calcaneus with a high PC1 score has a longer proximodistal length. Low PC1 scores generally represent the opposite condition. PC2 represents 1.32% of the variance. It is most strongly, although moderately, correlated with variables Ca4 (− 0.46) and Ca5 (0.49). Generally, a calcaneus with a high PC2 score has a tuber with a narrow caudal extremity and is thicker dorsoplantarly. Low PC2 scores generally represent the opposite conditions.

#### Astragalus

Results of principal component analysis include astragali attributed to *Tamquammys robustus* plotting within terrestrial and rock climbing species morphospace, which is distinct from that of arboreal and semiaquatic species (Fig. 5b). *T. wilsoni* plots closest to unspecialized arboreal species. Principal component 1 (PC1) represents 96.37% of the variance. All variables show positive loading for PC1 (Additional file 1: Figure S5), and all are weakly positively correlated (0.26–0.33) with this component. Generally, an astragalus with a high PC1 score is longer proximodistally. Low PC1 scores generally represent the opposite condition. PC2 represents 1.37% of the variance. It is strongly negatively correlated (− 0.73) with variable As4 and moderately positively correlated (0.49) with variable As8. Generally, an astragalus with a high PC2 score has a short astragalar neck and a longer medial crest. Low PC2 scores generally represent the opposite conditions.

Overall, principal component 1 (PC 1) can be viewed as primarily a measure of proximodistal length of the bones. We could explain its dominant contribution to the total variance by a general uniformity of the astragalocalcaneal morphology among studied taxa; thus a large influence of size even on standardized measurements (see Methods).

## Discussion

### Phylogenetic position of *Tamquammys* and the antiquity of ctenodactyloid rodents

The phylogenetic position of *Tamquammys* within Rodentia is very basal according to Vianey-Liaud and Marivaux [30]. It is in polytomy with a clade composed of (*Tribosphenomys* + *Archetypomys*) + *Cocomys*, with Ischyromyiformes considered as more derived. However, its exact relationships to other basal ctenodactyloids, such as *Cocomys* (or *Euxmys*) are ambiguous, as *Tamquammys* has been often proposed as more derived [3, 31–33].

In a broader phylogenetic context *Tamquammys* is close to the rodentiaform (sensu Wyss and Meng [34]) *Tribosphenomys* (Fig. 2), which is regarded therein as a basal member of Simplicidentata (see Meng and Wyss [35]). Whether *Tribosphenomys* and, more broadly, Alagomyidae are ancestral to rodents or represent a specialized offshoot of basal Glires, is a matter of debate. Nevertheless, comprehensive phylogenetic analyses point to the basal position of ctenodactyloids within Rodentia [30] and Tamquammyidae within ctenodactyloids (see Marivaux et al. [32]: fig. 4).

From a paleontological perspective early rodent phylogeny is complicated by the fact that the fossil record of the Asian ctenodactyloids (earliest Eocene, Bumbanian ALMA; [3, 4] is predated by the North American ischyromyids, in particular the reithroparamyine lineage, appearing already in the late Paleocene (early Clarkforkian NALMA; [36, 37], about one million years earlier than the earliest ctenodactyloid rodents). Which of the two rodent groups, ctenodactyloids or ischyromyids, represents the most basal rodents is a challenging question. Wible et al. ([33]: fig. 23) regarded paramyines as more basal than Ctenodactyloidea, and Meng and Wyss [38] suggested *Acritoparamys* and *Paramys* as stem rodents (but see Meng et al. [39]). In the current paper we follow the most recent phylogenetic analysis of Vianey-Liaud and Marivaux ([30]: fig. 7), which places ischyromyiform rodents as more nested than *Tamquammys*, thus pointing towards Ctenodactyloidea as the most ancient true rodent lineage.

### The astragalocalcaneal morphology in *Tamquammys* – In search of rodent ancestral morphotype

Szalay [15], in a discussion on the rodent locomotor adaptations modeled the ancestral ‘protorodent’ morphotype on the North American *Paramys*, which at that time was undoubtedly the best known representative of early rodents. Accordingly, he suggested that the earliest rodents were adapted mostly arboreal (as proposed for *Paramys* already by Wood [40]), but had evolved from generally terrestrial ‘protoplacentals’. Further analyses regarded paramyines as more generalized in their locomotion abilities, with a mixture of arboreal and terrestrial adaptations, also including facultative burrowing [17, 41, 42].

*Tamquammys* shares with Szalay’s ([15]: table 2) ‘protorodent’ the lack of the calcaneofibular facet, a helical ectal facet, and a slightly ovoid (especially in *T. wilsoni*) and deep calcaneocuboid surface. Whether it exhibited the tibiofibular syndesmosis in the lower fibular joint is uncertain, although likely, as this feature was also present in *Rhombomylus* [39] and *Paramys* [17]. Moreover, *Tamquammys* probably had a tibial posterior process, which is indicated by an anteroposteriorly extension of the trochlear surface on the astragalus. Unlike Szalay’s ‘protorodent’, *Tamquammys* has a relatively symmetrical astragalar trochlea and lesser mediolateral extension of the astragalar head, but the trochlea symmetry is a highly variable character in different paramyine genera according to Rose and Chinnery [17], and thus of little value for phylogenetic inference. On balance, the astragalocalcaneal complex of *Tamquammys* apparently exhibits a more ancestral morphotype for the rodents than that of paramyines.

The morphology of the astragalocalcaneal complex of *Tamquammys* reveals more traits in common with the earliest Rodentiaformes (e.g. *Tribosphenomys*) and even more archaic groups of early-to-middle Paleocene Glires [6, 35, 43, 44] as well as *Purgatorius* than between the Rodentiaformes and Paramyines (see also Fig. 5).

Further, the structure of the astragalocalcaneal complex in *Tamquammys* displays some plesiomorphic features regarded by Szalay and Decker [21] as ancestral for early Paleocene Eutheria (exemplified therein by *Protoungulatum*, *Procerberus*, and *Plesiadapis*). These features are: an oblique calcaneocuboid facet, the ectal facet positioned askew (inclined 35–40° to the long axis of the bone), the presence of the anterior plantar tubercle, the separate astragalar sustentacular facet, and a wide astragalar head, laterally thicker and rotated slightly dorsolaterally. Incidentally, most of these traits are poorly manifested in the late Cretaceous placental *Zalambdalestes*, which was regarded by Kielan-Jaworowska [45] as quite specialized. Apart from the above mentioned plesiomorphies, *Tamquammys* displays several important derived characters, such as some elongation of the calcaneal body, a deep, straight-ridged, and relatively long astragalar trochlea, the peroneal process midway down the calcaneocuboid junction and the sustentaculum tali; additionally the astragalus lacks an astragalar canal. The rotation axis at the astragalocalcaneal articulation is oblique (another primitive character of Szalay and Decker [21], same as in most rodents, but this obliquity is nonetheless lesser than in the early Paleocene Eutheria studied by Szalay and Decker [21]).

The question of the ancestral condition of the tarsal joint and locomotor adaptations in the earliest Glires remains open (given the uncertain adaptations in *Tribosphenomys*; Fig. 5), although the morphology of the astragalocalcaneal complex in *Tamquammys* suggests terrestriality, not arboreality, as a point of departure for Glires. Furthermore, *Tamquammys* groups rather close in the morphospace with the basal primate *Purgatorius* (Fig. 5), and in the latter the arboreal adaptations in the astragalocalcaneal complex are relatively weakly expressed. This may suggest that a more generalized foot structure was indeed characteristic of early Euarchontoglires. Beginning with the early middle Eocene, we observe the increased variety of tarsal modifications in Rodentia, which in part contributed to the evolutionary success of the order, which continues up to modern times.

## Conclusions

*Tamquammys* is one of the earliest ctenodactyloid rodents known from the early Paleogene of Asia. Its astragalocalcaneal morphology and locomotor adaptations are analyzed here against a broader background of major rodent morphotypes. The crurotarsal joint shows great mobility in the parasagittal plane and some limitations in mediolateral direction, which suggests general cursorial/terrestrial specialization, especially well expressed in a larger species (*T. robustus*). The calcaneocuboid articular surface is markedly inclined, which also limits its mobility and points to a general cursorial specialization. *T. wilsoni* shows a better jumping ability indicated e.g., by a longer calcaneal body. The PCA analysis places *T. robustus* within terrestrial and rock climber species morphospace, whereas *T. wilsoni* clusters with unspecialized arboreal rodent species such as *Glis glis*, tupaiid scandentians, and *Purgatorius*, a putative ‘proto-primate’. Because the morphology of the astragalocalcaneal ensemble of *Tamquammys* does not depart far from that of the early Paleocene *Purgatorius*, our findings contribute to establishing the original ‘paleorodent’ morphotype as close to basal Euarchonta.

## Methods

### Material

The tarsal bones of *Tamquammys robustus* come from the lower Arshantan (AS-1) of Nuhetingboerhe, whereas those of *T. wilsoni* come from both the Arshantan and Irdinmanhan (IM-1) strata of Nuhetingboerhe and Huheboerhe. Both localities are in the eastern part of the Erlian Basin, Nei Mongol, China (see Wang et al. [9, 46] for details). We have allocated the tarsal bones to respective *Tamquammys* species on the basis of dental remains accompanying the bones in the same localities. In the sediment beds of the lower Arshanto Formation *Tamquammys* is the only known rodent, represented by two species (*T. robustus* and *T. wilsoni*). The morphology of the bones from the Irdin Manha Formation assigned to *T. wilsoni* is consistent with that of the tarsal bones of the same species yielded by the Arshantan strata. Mass estimate for both species are provided in Fostowicz-Frelik et al. ([26]: table S6). Comparative material comprises 10 extant and 2 fossil rodent species, one rodentiaform, 2 duplicidentate taxa, and 2 basal Euarchontoglires representing main ecomorphs (generalist, arboreal and rock climbers, fossorial, semiaquatic, ambulatory, and cursorial), and is listed in Additional file 1 (Table S1). All *Tamquammys* specimens are deposited in the collection of the Institute of Vertebrate Paleontology and Paleoanthropology of Chinese Academy of Sciences, Beijing (abbreviated IVPP).

### Measurements

We took 15 linear measurements on the calcaneus of 36 specimens and 12 linear measurements on the astragalus of 25 specimens; measurements are provided in Additional file 1: Tables S4–5. We analyzed quantitative data using JMP 8 (SAS Institute Inc.). To estimate the interspecific differences, we used boxplots (Additional file 1: Figures S2–S3); each boxplot displays the 10th, 25th, the median, 75th and 90th percentiles of variable.

### Principal component analysis

To assess our qualitative observations we run principal component analysis on the variance-covariance matrix using the singular value decomposition (SVD) algorithm. PCA analyses were conducted using PAST v. 2.17 [47]. Before running PCA we size-standardized linear measurements using the geometric mean transformation of the data following Mosimann and James [48]. To measure suitability of our data for PCA we used a Kaiser-Meyer-Olkin (KMO) Test for Sampling Adequacy (see Additional file 1: Table S6) implemented in Factor Analysis v. 10.8.03 (U. Lorenza Seva & P.J. Ferrando, Universitat Rovira i Virgili, Tarragona, Spain). For eigenvalue, component loadings and percentage variance for each principal component, see Additional file 1: Table S7.

### Terminology and abbreviations

The terminology of tarsal elements follows Gladman et al. [19] with some modifications from Evans [49] and Szalay [15]. The measurements and anatomical terms used in the paper are provided in Additional file 1: Figure S1 and listed on Tables S2–S3.

## Additional file

Additional file 1: **Table S1.** Specimens examined in morphological analyses. **Table S2.** Guide to the bone measurements – calcaneus. **Table S3.** Guide to the bone measurements – astragalus. **Figure S1.** Measurements of the tarsal elements shown at the right calcaneus (A, B) and right astragalus (C, D) of *Tamquammys robustus* (IVPP coll. V24136.1 and IVPP coll. V24136.4, respectively) from the Arshanto Formation (early Eocene) of Nuhetingboerhe section, Erlian Basin, Nei Mongol, China. **Figure S2.** Ratios for calcaneal measurements. A, slenderness ratio (CW/CL); B, calcaneal load arm (CBL/CL); C tuber proportions (TCW/TCdp); D, relative length of the ectal facet (CEL/CL); E, proportions of the calcaneocuboid facet (CaCuW/CaCuL). Abbreviations: A.te, *Arvicola terrestris*; C.cr, *Cricetus cricetus*; C.lud, *Cynomys ludovicianus*; G.gl, *Glis glis*; G.el, *Gomphos elkema*; M.ma, *Marmota marmota*; M.mu, *Mus musculus*; O.pa, *Ochotona pallasi*; O.zi, *Ondatra zibethicus*; Par., paramyine rodent; Pur, *Purgatorius*; R.no, *Rattus norvegicus*; S.vu, *Sciurus vulgaris*; T.gl, *Tupaia glis*; T.mi, *Tribosphenomys minutus*; T.ro, *Tamquammys robustus*; T.wi, *Tamquammys wilsoni*. Colors: red for rodents; yellow for Rodentiaformes; green for stem duplicidentate and lagomorph; blue for Euarchonta (basal primate and Scandentia). **Figure S3.** Ratios for astragalar measurements. A, trochlear ratio (TW/AL); B, neck ratio (NL/AL); C head proportions (HW/NL); D, trochlear crests ratio (MCL/LCL); abbreviations as in Figure S2. **Table S4.** Measurements of calcaneus of *Tamquammys robustus*, *T. wilsoni*, and comparative taxa (in mm). **Table S5.** Measurements of astragalus of *Tamquammys robustus* and *T. wilsoni* and comparative taxa (in mm). **Table S6.** Results of Kaiser-Meyer-Olkin (KMO) Test for sampling adequacy. **Table S7.** Eigenvalues for calcaneus (A) and astragalus (B) PCA analysis. **Figure S4.** PCA loadings for the calcaneus. Loadings are for variables described in Table S2 (Ca1–15). **Figure S5.** PCA loadings for the astragalus. Loadings are for variables described in Table S3 (As1–12). (DOCX 1745 kb)
