# Supplementary material for: Oldest ctenodactyloid tarsals from the Eocene of China and evolution of locomotor adaptations in early rodents
Source: BMC Evol Biol. 2018 Oct 4;18:150. doi: 10.1186/s12862-018-1259-1 (PMC6172738; doi:10.1186/s12862-018-1259-1)

**Additional files (Figs S1–S5 and Tabs S1–S7)**

**Oldest ctenodactyloid tarsals from the Eocene of China and evolution of locomotor adaptations in early rodents**

Łucja Fostowicz-Frelik^*1,2^, Qian Li^1^, Xijun Ni^1^

^1^Key Laboratory of Vertebrate Evolution and Human Origins, Institute of Vertebrate Paleontology and Paleoanthropology, Chinese Academy of Sciences, 100044 Beijing, People’s Republic of China (liqian@ivpp.ac.cn; nixijun@ivpp.ac.cn)

^2^Institute of Paleobiology, Polish Academy of Sciences, PL 00-818 Warszawa, Poland; (lfost@twarda.pan.pl) [*to whom correspondence should be addressed]

**Table S1** Specimens examined in morphological analyses

| **Taxon** | **Collection number** | **Status: (E) extant, (F) fossil** |
| --- | --- | --- |
| *Arvicola terrestris* | ISEZ M/13423 | **(E)** |
| *Cricetus cricetus* | ISEZ M/13323 | **(E)** |
| *Cynomys ludovicianus* | IVPP-coll. 81-33A-2-18 | **(E)** |
| *Glis glis* | ISEZ M/8510/88  ISEZ M/12110 | **(E)**  **(E)** |
| *Gomphos elkema* | uncataloged IVPP coll. | **(F)**, Nuhetingboerhe, Erlian Basin, Nei Mongol, China |
| *Marmota marmota* | NX-coll.^1^ | **(E)** |
| *Mus musculus* | ISEZ M/13329 | **(E)** |
| *Ochotona pallasi* | ISEZ M/2984/66 | **(E)** |
| *Ondatra* *zibethicus* | NX-coll | **(E)** |
| Paramyinae indet. | Calcaneus R; uncataloged IVPP coll. | **(F)** Irdin Manha Fm. Erlian Basin, Nei Mongol, China |
| *Paramys* sp. | USNM 491851 astragalus R; literature data^2^ | **(F)** |
| *Purgatorius* sp. | UCMP 197509, L astragalus  UCMP 197517, R calcaneus  Literature data^3^ | **(F)** Paleocene (late Puercan) Garbani Channel fauna; localities in NE Montana, USA |
| *Rattus norvegicus* | IVPP coll. 417-4-7-4 | **(E)** |
| *Sciurotamias* *davidianus* | IVPP-coll. 780-33A-1-16 | **(E)** |
| *Sciurus vulgaris* | ISEZ M/12119  ISEZ M/13322  ISEZ M/13337 | **(E)**  **(E)**  **(E)** |
| *Tamquammys robustus* | IVPP coll. V24136.1-3 R calcanei  IVPP coll. V24136.4-6 R astragalus and two L astragali, respectively | **(F)** Nuhetingboerhe, Erlian Basin, Nei Mongol, China; Arshanto Fm. (late early to early middle Eocene) |
| *Tamquammys wilsoni* | IVPP coll. V24137.1-3 R calcaneus and L calcanei  IVPP coll. V24137.4-6 L astragali  IVPP coll. V24138 R calcaneus  IVPP coll. V24139 L astragalus  IVPP coll. V24140 L calcaneus  IVPP coll. V24141 L astragalus  IVPP coll. V24142 L astragalus | Nuhetingboerhe, Erlian Basin, Nei Mongol, China; Arshanto Fm. (late early to early middle Eocene)  Huheboerhe (H-2), Nei Mongol, China; Irdin Manha Fm. middle Eocene  Huheboerhe (H-2A), Nei Mongol, China, Irdin Manha Fm. middle Eocene |
| *Tribosphenomys minutus* | uncataloged IVPP coll. | **(F)** Subeng, Erlian Basin, China, Late Paleocene |
| *Tupaia glis* | NX-coll. | **(E)** |

^1^ Ni Xijun comparative collection at IVPP (NX-coll.), ^2^ Data from Rose and Chinnery (2004), ^3^ Data from Chester et al. (2015); ISEZ, Institute of Systematics and Evolution of Animals, Polish Academy of Sciences, Kraków, Poland; IVPP, Institute of Vertebrate Paleontology and Paleoanthropology, Chinese Academy of Sciences, Beijing, People’s Republic of China

**Table S2** Guide to the bone measurements – calcaneus

| Measurement No. | Description | Abbreviation | Figure |
| --- | --- | --- | --- |
| 1 | Total bone length | CL | FigS1A |
| 2 | Maximum bone width, measured from the medial tip of the sustentaculum tali to the lateral tip of the peroneal process | CW | FigS1A |
| 3 | Length of the calcaneal body | CBL | FigS1A |
| 4 | Width of the calcaneal tuber, measured at its caudal extremity | TCW | FigS1A |
| 5 | Dorsoplantar dimension of the calcaneal tuber, measured at its caudal extremity | TCdp | FigS1B |
| 6 | Length of the calcaneal eminence | EML | FigS1B |
| 7 | Length of the ectal facet | CEL | Fig1A |
| 8 | Width of the ectal facet at its cranial margin | EWCr | Fig1A |
| 9 | Width of the ectal facet in its mid-length | EWm-l | Fig1B |
| 10 | Maximum width of the calcaneal eminence | EW | Fig1A |
| 11 | Maximum dorsoplantar dimension of the bone, measured from the dorsalmost point of the calcaneal eminence to the plantarmost point of the anterior plantar tubercle | DP | Fig1B |
| 12 | Width of the calcaneal body, measured from the medialmost point of the calcaneal body (anteriorly to the sustentaculum tali) to the lateralmost point of the peroneal tubercle | CBW | Fig1A |
| 13 | Width of the calcaneocuboid facet, the maximum mediolateral dimension of the articular facet | CaCuW | Fig1A |
| 14 | Dorsoplantar length of the calcaneocuboid facet | CaCuL | Fig1B |
| 15 | Width of the calcaneal tuber in mid-length | TCWm-l | Fig1A |

**Table S3** Guide to the bone measurements – astragalus

| Measurement No. | Description | Abbreviation | Figure |
| --- | --- | --- | --- |
| 1 | Total bone length | AL | Fig1C |
| 2 | Total width of the astragalar body | AW | Fig1C |
| 3 | Total trochlea width | TW | Fig1C |
| 4 | Length of the astragalar neck | NL | Fig1C |
| 5 | Mediolateral length of the astragalar head | HL | Fig1C |
| 6 | Dorsoplantar width of the astragalar head | HW | Fig1C |
| 7 | Length of the lateral crest | LCL | Fig1C |
| 8 | Length of the medial crest | MCL | Fig1C |
| 9 | Width of the astragalar ectal facet | AEW | Fig1D |
| 10 | Length of the astragalar ectal facet | AEL | Fig1D |
| 11 | Length of the sustentacular facet | SL | Fig1D |
| 12 | Width of the sustentacular facet | SW | Fig1D |


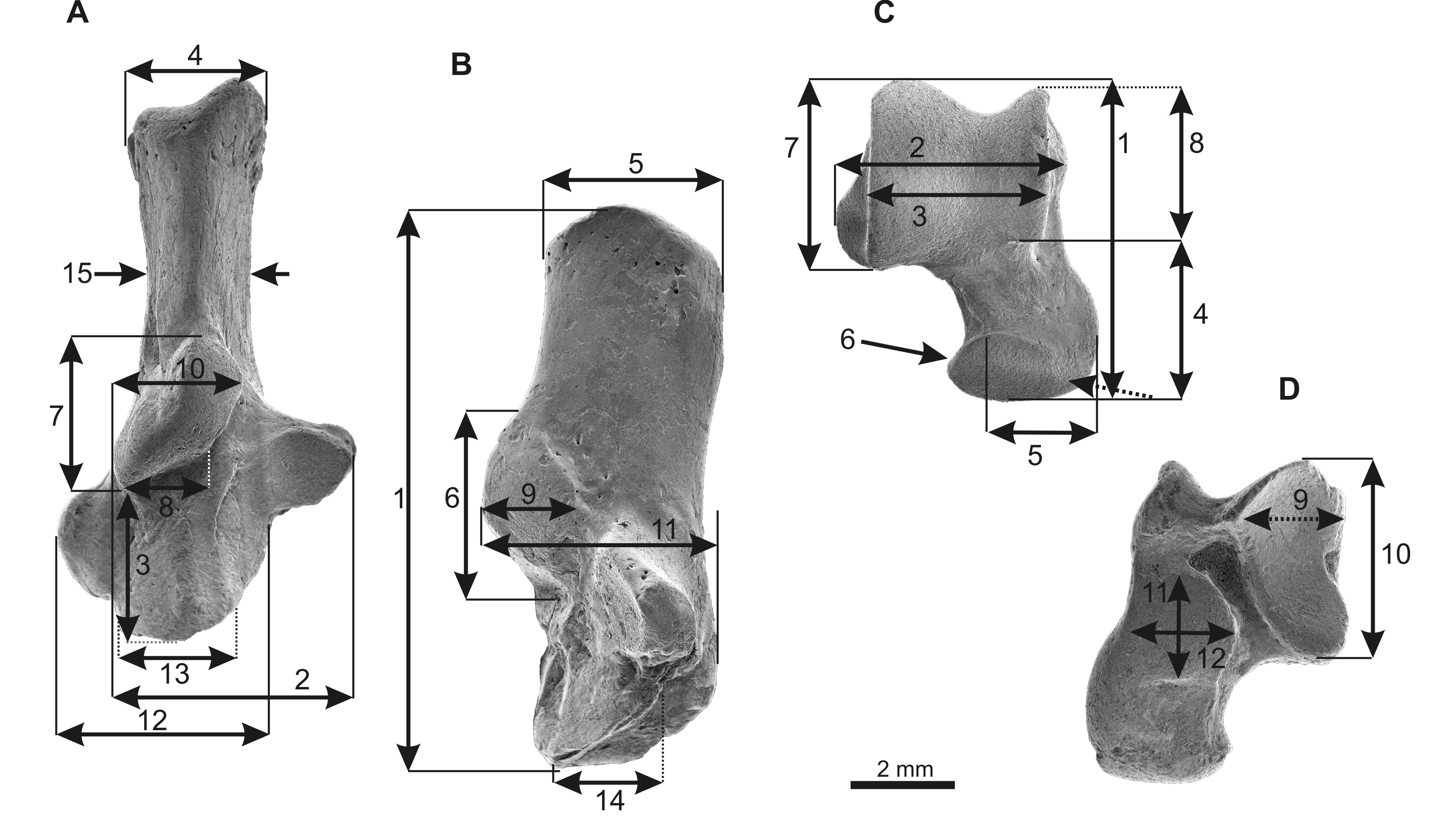


**Fig. S1** Measurements of the tarsal elements shown at the right calcaneus (A, B) and right astragalus (C, D) of *Tamquammys robustus* (IVPP coll. V24136.1 and IVPP coll. V24136.4, respectively) from the Arshanto Formation (early Eocene) of Nuhetingboerhe section, Erlian Basin, Nei Mongol, China.


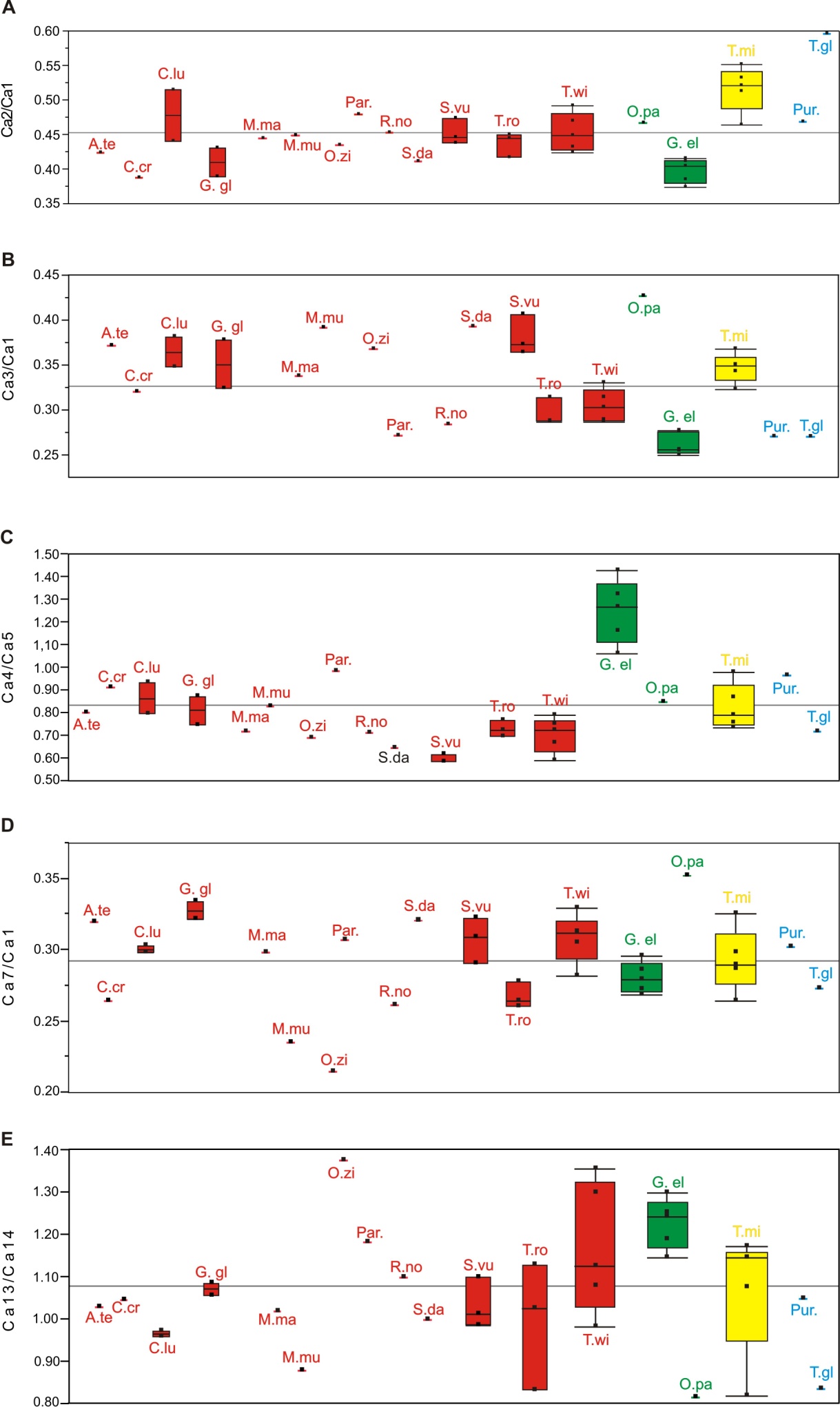


**Fig. S2** Ratios for calcaneal measurements. A, slenderness ratio (CW/CL); B, calcaneal load arm (CBL/CL); C tuber proportions (TCW/TCdp); D, relative length of the ectal facet (CEL/CL); E, proportions of the calcaneocuboid facet (CaCuW/CaCuL). Abbreviations: A.te, *Arvicola terrestris*; C.cr, *Cricetus cricetus*; C.lud, *Cynomys ludovicianus*; G.gl, *Glis glis*; G.el, *Gomphos elkema*; M.ma, *Marmota marmota*; M.mu, *Mus musculus*; O.pa, *Ochotona pallasi*; O.zi, *Ondatra zibethicus*; Par., paramyine rodent; Pur, *Purgatorius*; R.no, *Rattus norvegicus*; S.vu, *Sciurus vulgaris*; T.gl, *Tupaia glis*; T.mi, *Tribosphenomys minutus*; T.ro, *Tamquammys robustus*; T.wi, *Tamquammys wilsoni*. Colors: red for rodents; yellow for Rodentiaformes; green for stem duplicidentate and lagomorph; blue for Euarchonta (basal primate and Scandentia).


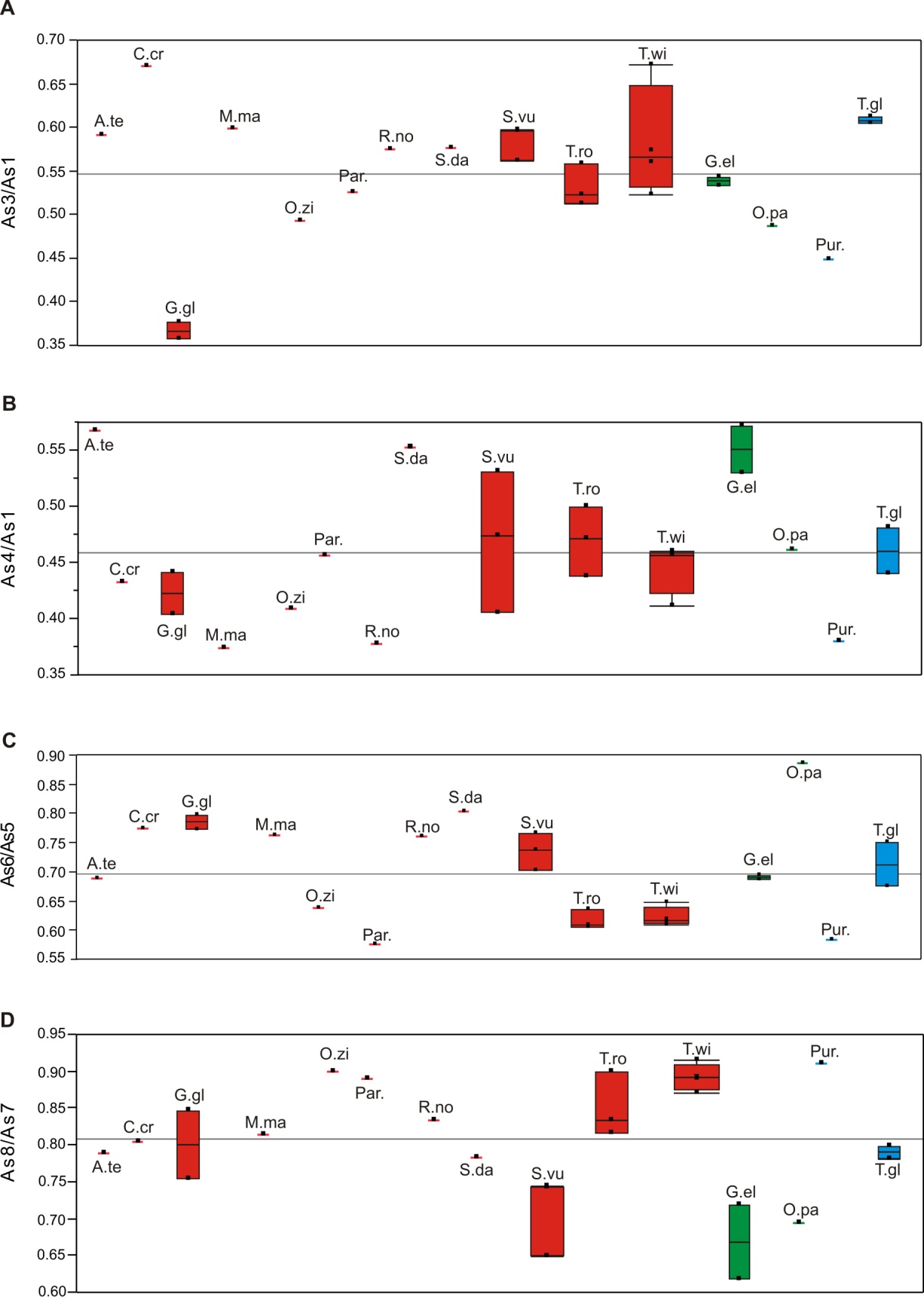


**Fig. S3** Ratios for astragalar measurements. A, trochlear ratio (TW/AL); B, neck ratio (NL/AL); C head proportions (HW/NL); D, trochlear crests ratio (MCL/LCL); abbreviations as in Fig.S2.

**Table S4**

Measurements of calcaneus of *Tamquammys robustus*, *T*. *wilsoni*, and comparative taxa (in mm)

| Measurement | 1 | 2 | 3 | 4 | 5 | 6 | 7 | 8 | 9 | 10 | 11 | 12 | 13 | 14 | 15 |
| --- | --- | --- | --- | --- | --- | --- | --- | --- | --- | --- | --- | --- | --- | --- | --- |
| *Tamquammys robustus* | | | | | | | | | | | | | | | |
| V24136.1  V24136.2  V24136.3 | 10.62  9.83  10.62 | 4.77  4.09  4.72 | 3.05  3.08  3.04 | 2.47  2.46  2.45 | 3.43  3.20  3.53 | 3.0  2.75  2.98 | 2.76  2.60  2.95 | 1.67  1.63  1.82 | 1.56  1.43  1.36 | 2.19  2.0  2.11 | 4.04  4.50  4.38 | 4.38  3.73  4.14 | 2.57  2.30  2.20 | 2.51  2.04  2.65 | 1.89  1.85  1.90 |
| *Tamquammys wilsoni* | | | | | | | | | | | | | | | |
| V24137.1  V24137.2  V24137.3  V24138.1  V24140 | 5.67  5.57  5.86  5.92  5.51 | 2.54  2.36  2.53  2.78  2.71 | 1.62  1.75  1.69  1.79  1.82 | 1.45  1.29  1.17  1.73  1.19 | 1.94  1.79  1.99  2.19  1.78 | 1.79  1.77  1.95  2.07  1.59 | 1.77  1.70  1.93  1.85  1.55 | 0.99  1.09  0.95  1.20  0.80 | 0.74  0.88  1.0  0.95  0.80 | 1.15  1.06  1.0  1.20  1.05 | 2.60  2.36  2.52  2.60  2.25 | 3.27  2.14  2.43  3.32  2.24 | 1.43  1.64  1.96  1.68  1.84 | 1.46  1.46  1.45  1.56  1.42 | 1.01  1.01  1.0  1.23  1.03 |
| *Tribosphenomys minutus* | | | | | | | | | | | | | | | |
| IVPP uncat. 1  IVPP uncat. 2  IVPP uncat. 3  IVPP uncat. 4  IVPP uncat. 5 | 2.52  2.95  2.91  2.83  2.48 | 1.34  1.51  1.35  1.56  1.29 | 0.88  1.01  0.94  0.99  0.91 | 0.78  0.86  0.77  0.86  0.812 | 0.90  1.17  1.02  1.09  0.83 | 0.77  0.96  0.81  0.84  0.78 | 0.73  0.96  0.77  0.81  0.74 | 0.37  0.45  0.49  0.40  0.41 | 0.62  0.69  0.55  0.60  0.45 | 0.76  0.78  0.69  0.71  0.69 | 1.34  1.70  1.52  1.61  1.40 | 1.26  1.34  1.25  1.30  1.25 | 0.41  0.99  0.87  0.80  0.69 | 0.35  1.21  0.81  0.70  0.69 | 0.56  0.61  0.57  0.62  0.58 |
| *Gomphos elkema* | | | | | | | | | | | | | | | |
| IVPP uncat. 1  IVPP uncat. 2  IVPP uncat. 3  IVPP uncat. 4  IVPP uncat. 5 | 21.0  22.05  22.34  23.07  24.16 | 8.62  8.90  8.36  9.57  9.30 | 5.34  6.09  5.57  6.33  6.17 | 6.90  6.35  7.32  7.73  7.88 | 5.42  5.96  6.26  5.83  5.50 | 7.29  7.47  7.33  7.11  7.40 | 6.0  5.92  6.22  6.81  6.57 | 3.93  3.56  3.45  3.48  4.30 | 3.43  3.33  3.41  2.76  3.50 | 4.17  4.74  4.48  4.41  4.88 | 9.16  9.02  9.64  9.66  10.51 | 6.57  7.11  7.53  6.90  7.42 | 6.07  6.41  6.38  5.52  6.61 | 5.11  5.13  4.92  4.83  5.33 | 4.71  4.45  5.13  4.65  4.90 |
| *Ochotona pallasi* | | | | | | | | | | | | | | | |
| ISEZ M/2984/66 | 8.19 | 3.82 | 3.49 | 2.12 | 2.51 | 3.21 | 2.88 | 0.50 | 1.29 | 2.07 | 2.95 | 3.01 | 2.34 | 2.88 | 2.07 |
| *Arvicola terrestris* | | | | | | | | | | | | | | | |
| ISEZ M/13423 | 5.54 | 2.35 | 2.06 | 1.67 | 2.09 | 1.76 | 1.77 | 1.09 | 0.99 | 1.30 | 2.08 | 2.32 | 1.88 | 1.83 | 1.52 |
| *Cricetus cricetus* | | | | | | | | | | | | | | | |
| ISEZ M/13323 | 10.48 | 4.06 | 3.35 | 2.88 | 3.16 | 2.84 | 2.77 | 1.97 | 1.98 | 2.06 | 3.13 | 4.09 | 2.88 | 2.76 | 2.14 |
| *Cynomys ludovicianus* | | | | | | | | | | | | | | | |
| IVPP-coll. 81-33A-2-18 | 11.61  12.04 | 5.98  5.30 | 4.03  4.59 | 3.63  4.40 | 4.57  4.72 | 3.46  3.71 | 3.46  3.65 | 2.11  1.92 | 2.10  2.01 | 2.81  2.78 | 4.78  5.29 | 6.12  4.93 | 3.60  3.45 | 3.71  3.60 | 2.28  2.29 |
| *Glis glis* | | | | | | | | | | | | | | | |
| ISEZ M/8510/88  ISEZ M/12110 | 5.99  6.65 | 2.58  2.59 | 2.26  2.15 | 1.44  1.63 | 1.93  1.17 | 2.07  2.44 | 2.0  2.14 | 1.02  1.09 | 1.16  1.21 | 1.09  1.16 | 2.22  2.21 | 2.17  2.79 | 1.70  1.75 | 1.57  1.66 | 1.06  1.02 |
| *Marmota marmota* | | | | | | | | | | | | | | | |
| IVPP NX-coll. | 20.18 | 8.98 | 6.82 | 5.63 | 7.84 | 6.50 | 6.01 | 3.85 | 3.67 | 4.28 | 8.40 | 8.37 | 5.04 | 4.96 | 3.95 |
| *Mus musculus* | | | | | | | | | | | | | | | |
| ISEZ M/13329 | 2.94 | 1.32 | 1.15 | 0.68 | 0.82 | 0.73 | 0.69 | 0.44 | 0.44 | 0.63 | 1.04 | 1.24 | 0.85 | 0.97 | 0.51 |
| *Ondatra zibethicus* | | | | | | | | | | | | | | | |
| IVPP NX-coll. | 17.26 | 7.50 | 6.35 | 4.35 | 6.31 | 3.87 | 3.71 | 2.61 | 2.99 | 4.52 | 6.36 | 6.47 | 4.81 | 3.50 | 3.44 |
| *Paramyinae* indet. | | | | | | | | | | | | | | | |
| IVPP ucat. | 26.0 | 12.45 | 7.05 | 7.57 | 7.68 | 8.12 | 7.97 | 4.81 | 3.74 | 7.20 | 11.49 | 10.89 | 7.67 | 6.50 | 4.80 |
| *Rattus norvegicus* | | | | | | | | | | | | | | | |
| IVPP coll. 417-4-7-4 | 7.97 | 3.61 | 2.26 | 1.94 | 2.72 | 2.73 | 2.08 | 1.19 | 1.32 | 1.61 | 3.02 | 3.27 | 2.04 | 1.86 | 1.64 |
| *Sciurotamias davidianus* | | | | | | | | | | | | | | | |
| IVPP-coll. 780-33A-1-16 | 9.75 | 4.01 | 3.83 | 2.18 | 3.37 | 3.22 | 3.13 | 1.69 | 1.49 | 2.10 | 3.47 | 4.42 | 2.49 | 2.50 | 1.91 |
| *Sciurus vulgaris* | | | | | | | | | | | | | | | |
| ISEZ M/13322  ISEZ M/13337  ISEZ M/12119 | 10.74  10.53  11.10 | 4.79  4.99  4.86 | 4.36  3.92  4.04 | 2.39  2.45  2.42 | 3.86  3.98  4.13 | 3.80  3.29  3.21 | 3.32  3.40  3.22 | 1.82  2.18  1.93 | 1.71  1.99  1.80 | 1.88  2.15  1.98 | 4.38  4.59  4.46 | 4.22  4.18  3.67 | 2.67  2.57  2.88 | 2.64  2.61  2.63 | 1.54  1.55  1.48 |
| *Tupaia glis* | | | | | | | | | | | | | | | |
| IVPP NX-coll. | 6.96 | 4.15 | 1.88 | 1.73 | 2.41 | 1.97 | 1.90 | 1.19 | 1.25 | 1.44 | 2.69 | 2.85 | 1.46 | 1.75 | 1.50 |

**Table S5** Measurements of astragalus of *Tamquammys robustus* and *T*. *wilsoni* and comparative taxa (in mm)

| Measurement | 1 | 2 | 3 | 4 | 5 | 6 | 7 | 8 | 9 | 10 | 11 | 12 |
| --- | --- | --- | --- | --- | --- | --- | --- | --- | --- | --- | --- | --- |
| *Tamquammys robustus* | | | | | | | | | | | | |
| V24136.4  V24136.5  V24136.6 | 6.91  6.30  5.82 | 4.55  4.36  4.34 | 3.54  3.29  3.25 | 3.02  3.15  2.74 | 3.33  3.02  2.86 | 2.01  1.92  1.74 | 3.97  3.50  3.46 | 3.31  3.15  2.82 | 2.67  2.08  2.34 | 3.13  3.26  3.20 | 2.14  2.24  2.02 | 1.81  1.64  1.71 |
| *Tamquammys wilsoni* | | | | | | | | | | | | |
| V24137.4  V24137.5  V24141  V24142  V24139 | 3.77  4.17  3.12  3.23  3.21 | 2.52  2.64  2.26  2.06  1.98 | 2.11  2.18  1.60  1.66  1.84 | 1.72  1.90  1.31  1.40  1.32 | 1.89  1.97  1.66  1.60  1.61 | 1.21  1.20  1.0  0.98  0.99 | 2.15  2.27  1.94  1.97  2.01 | 1.87  2.02  1.80  1.84  1.84 | 1.49  1.29  1.02  1.20  1.18 | 2.45  2.33  1.73  1.53  1.76 | 1.60  1.49  0.81  0.82  1.18 | 1.27  1.07  1.32  1.17  0.81 |
| *Gomphos elkema* | | | | | | | | | | | | |
| IVPP uncat (12170-1)  IVPP uncat (12099-1) | 12.23  12.92 | 8.13  8.70 | 6.52  7.03 | 6.49  7.41 | 6.08  6.81 | 4.22  4.67 | 7.3  7.59 | 4.51  5.45 | 4.42  4.71 | 6.79  7.53 | 5.48  5.04 | 3.72  3.95 |
| *Ochotona pallasi* | | | | | | | | | | | | |
| ISEZ M/2984/66 | 4.88 | 2.76 | 2.38 | 2.25 | 2.03 | 1.80 | 2.97 | 2.06 | 1.29 | 2.72 | 1.82 | 1.28 |
| *Arvicola terrestris* | | | | | | | | | | | | |
| ISEZ M/13423 | 3.38 | 2.48 | 2.00 | 1.92 | 1.89 | 1.30 | 2.36 | 1.86 | 1.42 | 1.98 | 1.30 | 0.99 |
| *Cricetus cricetus* | | | | | | | | | | | | |
| ISEZ M/12119 | 5.37 | 3.93 | 3.60 | 2.32 | 2.52 | 1.95 | 3.21 | 2.58 | 2.01 | 2.91 | 1.99 | 1.26 |
| *Glis glis* | | | | | | | | | | | | |
| ISEZ M/8510/88  ISEZ N/12110 | 4.64  4.60 | 2.46  2.38 | 1.66  1.73 | 2.05  1.86 | 1.67  1.68 | 1.29  1.34 | 2.39  2.43 | 1.80  2.06 | 1.52  1.41 | 2.48  2.38 | 1.72  2.04 | 1.26  1.07 |
| *Marmota marmota* | | | | | | | | | | | | |
| IVPP NX coll. | 12.11 | 8.82 | 7.25 | 4.53 | 6.20 | 4.72 | 8.28 | 6.74 | 5.0 | 7.32 | 5.45 | 3.75 |
| *Ondatra zibethicus* | | | | | | | | | | | | |
| IVPP NX coll. | 10.70 | 7.11 | 5.27 | 4.37 | 5.21 | 3.32 | 6.0 | 5.40 | 4.83 | 5.30 | 4.86 | 2.53 |
| *Paramys* sp. | | | | | | | | | | | | |
| USNM 491851 | 11.5 | 8.11 | 6.04 | 5.25 | 6.45 | 3.71 | 7.11 | 6.32 | 5.68 | 8.00 | 5.27 | 4.71 |
| *Rattus norvegicus* | | | | | | | | | | | | |
| IVPP coll. 417-4-7-4 | 5.11 | 3.50 | 2.94 | 1.93 | 2.12 | 1.61 | 2.89 | 2.41 | 2.04 | 2.68 | 2.12 | 1.40 |
| *Sciurotamias davidianus* | | | | | | | | | | | | |
| IVPP-coll. 780-33A-1-16 | 5.92 | 4.08 | 3.41 | 3.27 | 2.94 | 2.36 | 3.68 | 2.88 | 2.20 | 3.46 | 2.78 | 1.88 |
| *Sciurus vulgaris* | | | | | | | | | | | | |
| ISEZ M/13322  ISEZ M/13337  ISEZ M/12119 | 7.23  7.13  6.84 | 4.78  4.59  4.32 | 4.06  4.25  4.09 | 2.93  3.38  3.63 | 3.79  3.66  3.79 | 2.79  2.80  2.66 | 4.56  4.72  4.41 | 3.38  3.06  3.28 | 2.60  2.82  2.78 | 3.76  4.04  3.61 | 2.68  3.14  3.30 | 2.05  1.82  1.97 |
| *Tupaia glis* | | | | | | | | | | | | |
| IVPP NX coll. | 4.05  4.07 | 2.88  2.83 | 2.45  2.49 | 1.78  1.96 | 2.04  2.09 | 1.53  1.41 | 2.53  2.51 | 2.02  1.96 | 1.59  1.71 | 2.46  2.12 | 2.24  2.0 | 1.09  0.87 |

**Table S6** Results of Kaiser-Meyer-Olkin (KMO) Test for sampling adequacy

| Calcaneus | 0.8979 (good) |
| --- | --- |
| Astragalus | 0.90136 (very good) |

**Table S7** Eigenvalues for calcaneus (A) and astragalus (B) PCA analysis

A

| PC | Eigenvalue | % variance |
| --- | --- | --- |
| 1 | 9.65722 | 96.6 |
| 2 | 0.13154 | 1.3158 |
| 3 | 0.0480915 | 0.48105 |
| 4 | 0.0444457 | 0.44458 |
| 5 | 0.0334508 | 0.3346 |
| 6 | 0.0221309 | 0.22137 |
| 7 | 0.0167797 | 0.16785 |
| 8 | 0.016072 | 0.13611 |
| 9 | 0.0083259 | 0.083283 |
| 10 | 0.0072261 | 0.072282 |
| 11 | 0.0053616 | 0.053632 |
| 12 | 0.0038230 | 0.038242 |
| 13 | 0.0022059 | 0.022066 |
| 14 | 0.0017349 | 0.017355 |
| 15 | 0.0011764 | 0.011768 |

B

| PC | Eigenvalue | % variance |
| --- | --- | --- |
| 1 | 7.23773 | 96.366 |
| 2 | 0.103047 | 1.372 |
| 3 | 0.0649602 | 0.8649 |
| 4 | 0.0320865 | 0.42721 |
| 5 | 0.0301298 | 0.40116 |
| 6 | 0.0120705 | 0.16071 |
| 7 | 0.010684 | 0.14225 |
| 8 | 0.0086644 | 0.11536 |
| 9 | 0.0063013 | 0.83899 |
| 10 | 0.0023774 | 0.031654 |
| 11 | 0.0014124 | 0.018805 |
| 12 | 0.0012294 | 0.01637 |

**Fig. S4** PCA loadings for the calcaneus. Loadings are for variables described in Table S2 (Ca1–15)


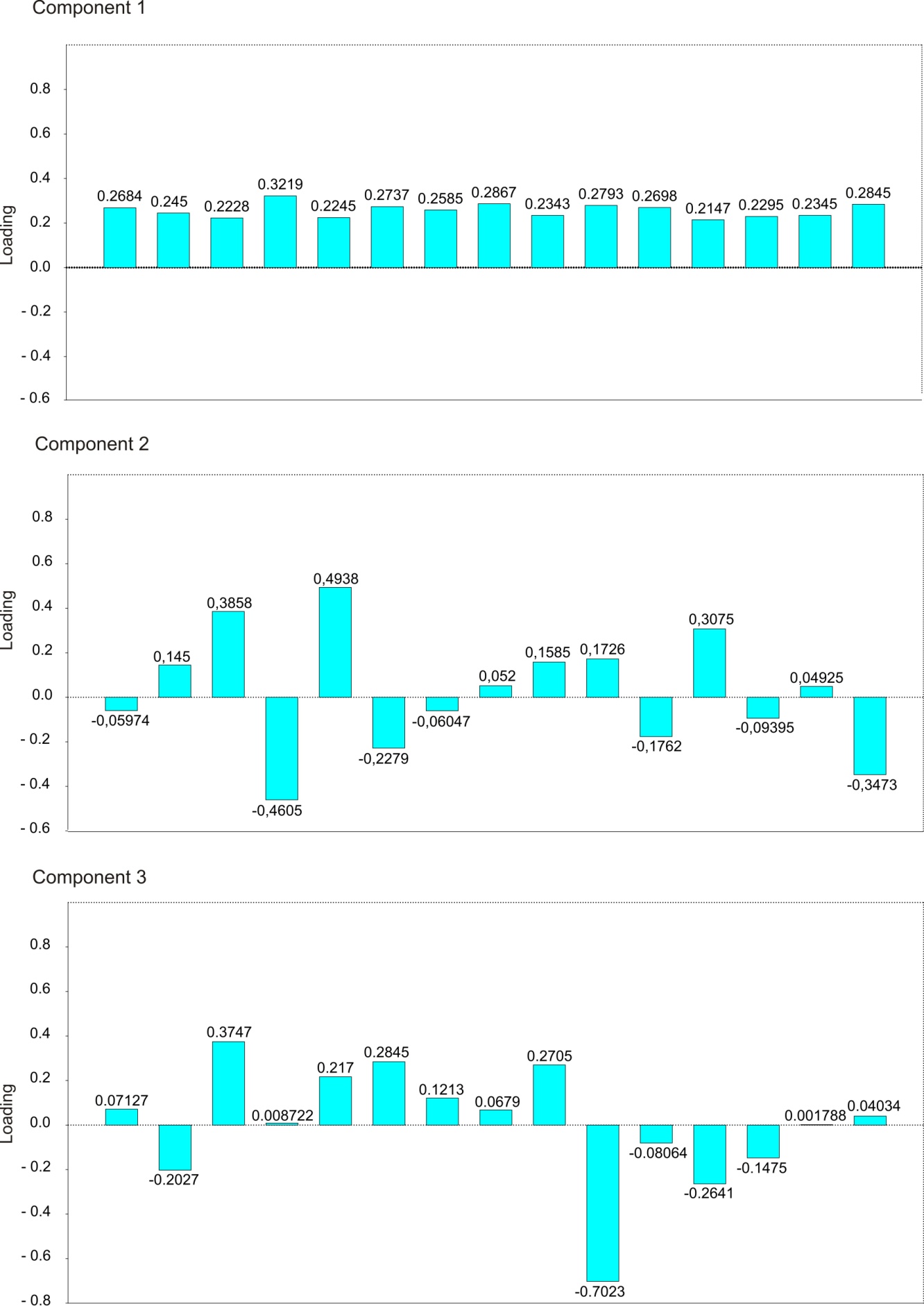


**Fig S5** PCA loadings for the astragalus. Loadings are for variables described in Table S3 (As1–12)


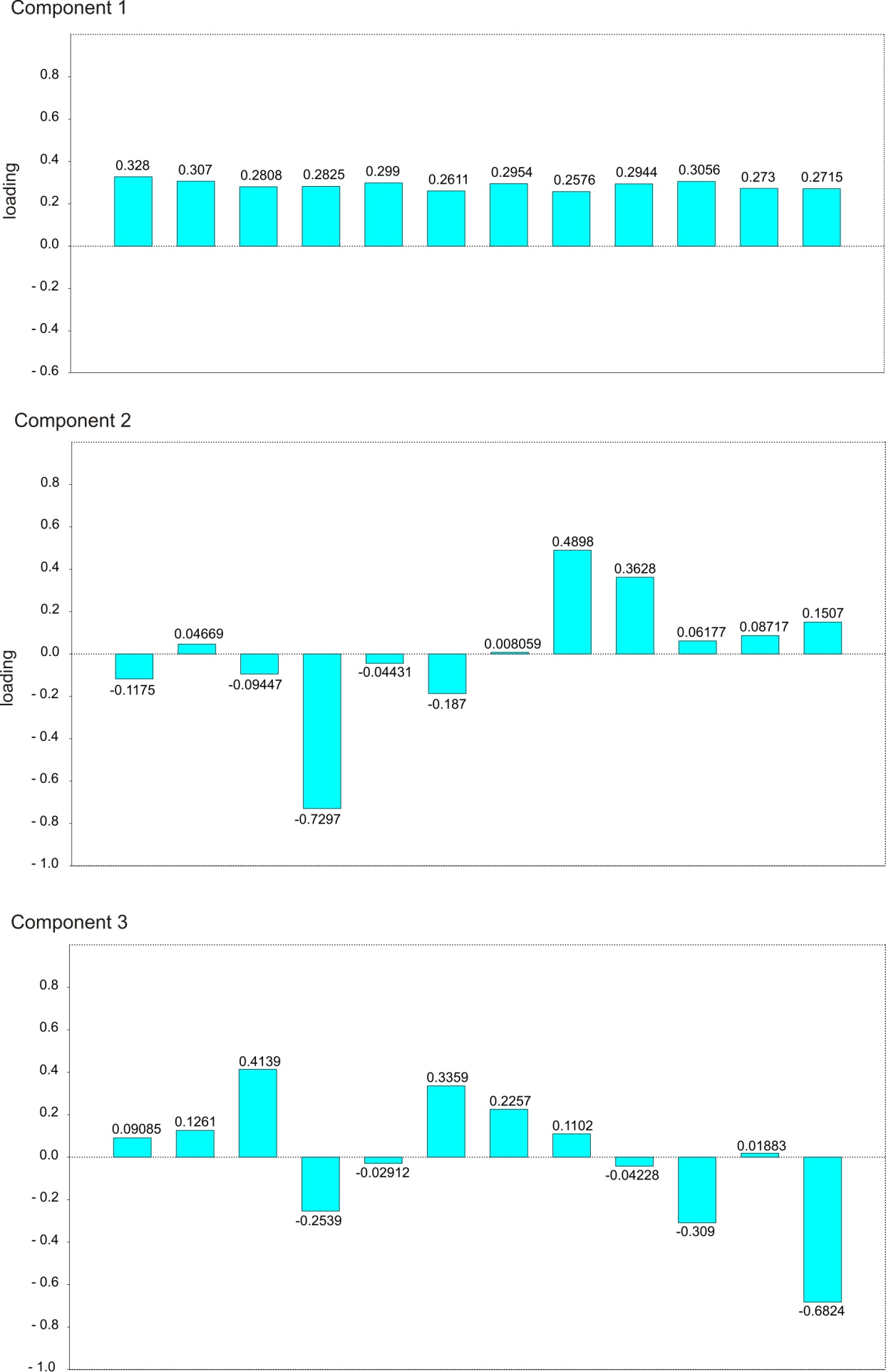

Supplement: Supplementary file 1 — Table S1. Specimens examined in morphological analyses. Table S2. Guide to the bone measurements – calcaneus. Table S3. Guide to the bone measurements – astragalus. Figure S1. Measurements of the tarsal elements shown at the right calcaneus (A, B) and right astragalus (C, D) of Tamquammys robustus (IVPP coll. V24136.1 and IVPP coll. V24136.4, respectively) from the Arshanto Formation (early Eocene) of Nuhetingboerhe section, Erlian Basin, Nei Mongol, China. Figure S2. Ratios for calcaneal measurements. A, slenderness ratio (CW/CL); B, calcaneal load arm (CBL/CL); C tuber proportions (TCW/TCdp); D, relative length of the ectal facet (CEL/CL); E, proportions of the calcaneocuboid facet (CaCuW/CaCuL). Abbreviations: A.te, Arvicola terrestris; C.cr, Cricetus cricetus; C.lud, Cynomys ludovicianus; G.gl, Glis glis; G.el, Gomphos elkema; M.ma, Marmota marmota; M.mu, Mus musculus; O.pa, Ochotona pallasi; O.zi, Ondatra zibethicus; Par., paramyine rodent; Pur, Purgatorius; R.no, Rattus norvegicus; S.vu, Sciurus vulgaris; T.gl, Tupaia glis; T.mi, Tribosphenomys minutus; T.ro, Tamquammys robustus; T.wi, Tamquammys wilsoni. Colors: red for rodents; yellow for Rodentiaformes; green for stem duplicidentate and lagomorph; blue for Euarchonta (basal primate and Scandentia). Figure S3. Ratios for astragalar measurements. A, trochlear ratio (TW/AL); B, neck ratio (NL/AL); C head proportions (HW/NL); D, trochlear crests ratio (MCL/LCL); abbreviations as in Figure S2. Table S4. Measurements of calcaneus of Tamquammys robustus, T. wilsoni, and comparative taxa (in mm). Table S5. Measurements of astragalus of Tamquammys robustus and T. wilsoni and comparative taxa (in mm). Table S6. Results of Kaiser-Meyer-Olkin (KMO) Test for sampling adequacy. Table S7. Eigenvalues for calcaneus (A) and astragalus (B) PCA analysis. Figure S4. PCA loadings for the calcaneus. Loadings are for variables described in Table S2 (Ca1–15). Figure S5. PCA loadings for the astragalus. Loadings are for variables d [file 12862_2018_1259_MOESM1_ESM.docx]
